# The endoplasmic reticulum proteostasis network profoundly shapes the protein sequence space accessible to HIV envelope

**DOI:** 10.1371/journal.pbio.3001569

**Published:** 2022-02-18

**Authors:** Jimin Yoon, Emmanuel E. Nekongo, Jessica E. Patrick, Tiffani Hui, Angela M. Phillips, Anna I. Ponomarenko, Samuel J. Hendel, Rebecca M. Sebastian, Yu Meng Zhang, Vincent L. Butty, C. Brandon Ogbunugafor, Yu-Shan Lin, Matthew D. Shoulders

**Affiliations:** 1 Department of Chemistry, Massachusetts Institute of Technology, Cambridge, Massachusetts, United States of America; 2 Department of Chemistry, Tufts University, Medford, Massachusetts, United States of America; 3 BioMicro Center, Massachusetts Institute of Technology, Cambridge, Massachusetts, United States of America; 4 Department of Ecology and Evolutionary Biology, Yale University, New Haven, Connecticut, United States of America; Fred Hutchinson Cancer Research Center, UNITED STATES

## Abstract

The sequence space accessible to evolving proteins can be enhanced by cellular chaperones that assist biophysically defective clients in navigating complex folding landscapes. It is also possible, at least in theory, for proteostasis mechanisms that promote strict quality control to greatly constrain accessible protein sequence space. Unfortunately, most efforts to understand how proteostasis mechanisms influence evolution rely on artificial inhibition or genetic knockdown of specific chaperones. The few experiments that perturb quality control pathways also generally modulate the levels of only individual quality control factors. Here, we use chemical genetic strategies to tune proteostasis networks via natural stress response pathways that regulate the levels of entire suites of chaperones and quality control mechanisms. Specifically, we upregulate the unfolded protein response (UPR) to test the hypothesis that the host endoplasmic reticulum (ER) proteostasis network shapes the sequence space accessible to human immunodeficiency virus-1 (HIV-1) envelope (Env) protein. Elucidating factors that enhance or constrain Env sequence space is critical because Env evolves extremely rapidly, yielding HIV strains with antibody- and drug-escape mutations. We find that UPR-mediated upregulation of ER proteostasis factors, particularly those controlled by the IRE1-XBP1s UPR arm, globally reduces Env mutational tolerance. Conserved, functionally important Env regions exhibit the largest decreases in mutational tolerance upon XBP1s induction. Our data indicate that this phenomenon likely reflects strict quality control endowed by XBP1s-mediated remodeling of the ER proteostasis environment. Intriguingly, and in contrast, specific regions of Env, including regions targeted by broadly neutralizing antibodies, display enhanced mutational tolerance when XBP1s is induced, hinting at a role for host proteostasis network hijacking in potentiating antibody escape. These observations reveal a key function for proteostasis networks in decreasing instead of expanding the sequence space accessible to client proteins, while also demonstrating that the host ER proteostasis network profoundly shapes the mutational tolerance of Env in ways that could have important consequences for HIV adaptation.

## Abstract 2

The host cell’s endoplasmic reticulum proteostasis network has a profound, constraining impact on the protein sequence space accessible to HIV’s envelope protein, which is a major target of the host’s adaptive immune system; in particular, upregulation of stringent quality control pathways appears to restrict the viability of destabilizing envelope variants.

## Introduction

Protein mutational tolerance is constrained by the biophysical properties of the evolving protein. Selection to maintain proper protein folding and structure purges a large number of otherwise possible mutations that could be functionally beneficial [1–5]. It is no surprise, then, that cellular proteostasis networks play a key role in defining the protein sequence space accessible to client proteins [6–17]. Much attention has been given to the phenomenon of chaperones increasing the sequence space accessible to their client proteins, likely by promoting the folding of protein variants with biophysically deleterious amino acid substitutions [7–11]. Most efforts in this area have focused specifically on how the activities of the heat shock proteins Hsp90 and Hsp70 can expand protein sequence space, in part owing to the availability of specific inhibitors that enable straightforward comparative studies of protein evolution in the presence versus the absence of folding assistance.

In contrast to chaperones increasing sequence space, one might anticipate that protein folding quality control factors would constrain the sequence space accessible to evolving client proteins. For example, promoting the rapid degradation and removal of slow-folding or aberrantly folded protein variants could cut off otherwise accessible evolutionary trajectories [16–18], especially if those variants might have still maintained some level of function if instead allowed to persist in the cellular environment. Unfortunately, efforts to understand the potential contributions of quality control in shaping protein sequence space are limited. This gap in understanding is particularly problematic because natural cellular mechanisms to remodel proteostasis networks function via stress-responsive transcription factors [19,20], rather than via inhibition or upregulation of individual chaperones. These transcription factors tune the levels of both chaperones and quality control mechanisms simultaneously. Such mechanisms may potentially compete in how they impact the sequence space of various evolving client proteins.

Here, we evaluated whether and how the unfolded protein response (UPR)–regulated endoplasmic reticulum (ER) proteostasis network influences the sequence space accessible to membrane proteins processed by the secretory pathway. In particular, we used chemical genetic control of the UPR to broadly modulate the composition of the ER proteostasis network, and then used deep mutational scanning (DMS) to assess how such perturbations alter accessible client protein sequence space. We chose human immunodeficiency virus-1 (HIV-1) envelope (Env), a trimeric surface glycoprotein that is folded and quality-controlled by the ER, as our model client protein. We selected Env because its rapid evolution during HIV infections plays a critical role in HIV developing drug and host cell antibody resistance [21–23]. Additionally, Env interacts extensively with various components of the ER proteostasis network, including the ER chaperones calnexin [24] and calreticulin [25], binding immunoglobulin protein (BiP) [26], and ER alpha-mannosidase to initiate ER-associated degradation (ERAD) [27,28], suggesting the strong potential for the host ER proteostasis network to shape Env’s accessible sequence space.

Importantly, recent work has revealed that the cellular proteostasis network can indeed impact the sequence space of not just endogenous client proteins, but also viral proteins that hijack their host’s proteostasis machinery [29–33]. This relationship has critical evolutionary and therapeutic implications, because mutational tolerance is directly associated with the ability of a virus to evade the host’s innate and adaptive immune responses, as well as antiviral drugs [34–40]. Early work in this area focused on how viruses like influenza and poliovirus hijack the host’s heat-shock-response-regulated cytosolic chaperones to enhance their mutational tolerance [29–31]. More recently, we discovered that host UPR-mediated upregulation of the ER proteostasis network increases the mutational tolerance of influenza A hemagglutinin specifically at febrile temperatures [32]. Aside from that hemagglutinin work, to our knowledge no comprehensive studies testing the influence of the ER proteostasis network on client protein evolution, whether viral or endogenous, are available.

In this study, we used chemical genetic tools to specifically induce the inositol-requiring enzyme-1/X-box binding protein-1 spliced (IRE1-XBP1s) and activating transcription factor 6 (ATF6) transcriptional arms of the UPR separately or in tandem [41]. This approach provided user-defined modulation of the composition of the host’s ER proteostasis network that mimics the cell’s natural stress response. We observed that the resulting distinct host environments caused a global decrease in Env mutational tolerance, particularly upon XBP1s-mediated enhancement of the ER proteostasis machinery. In addition, we observed that sites with different structural or functional roles responded differently to UPR upregulation. For example, conserved regions of Env exhibited an especially strong reduction in mutational tolerance, while a number of sites targeted by broadly neutralizing antibodies displayed an increase in mutational tolerance.

This work demonstrates for the first time, to our knowledge, that combined upregulation of chaperones and quality control factors can actually greatly decrease the mutational tolerance of a client protein. It also provides experimental evidence that the host ER proteostasis network profoundly shapes the protein sequence space available to viral membrane proteins and, critically, that the details of the interaction vary from one protein to another—and even within different regions of the same protein.

## Results

### Chemical genetic control of ER proteostasis network composition during HIV infection

We began by generating a cell line in which HIV could robustly replicate and we could chemically induce the UPR’s IRE1-XBP1s and ATF6 transcriptional responses separately or simultaneously, in an ER stress-independent manner. We sought ER stress-independent induction of these transcription factors rather than global stress-mediated UPR induction, owing to the pleiotropic effects of chemical stressors and the non-physiologic, highly deleterious consequences of inducing high levels of protein misfolding in the secretory pathway [19,32,41,42]. We selected the IRE1-XBP1s and ATF6 arms of the UPR for chemical control because, in contrast to the protein-kinase-R-like ER kinase arm of the UPR that functions largely through translational attenuation, they are the key pathways responsible for defining levels of ER chaperones and quality control factors [20,41,43] likely to influence Env folding, degradation, and secretion.

**Fig 1:**
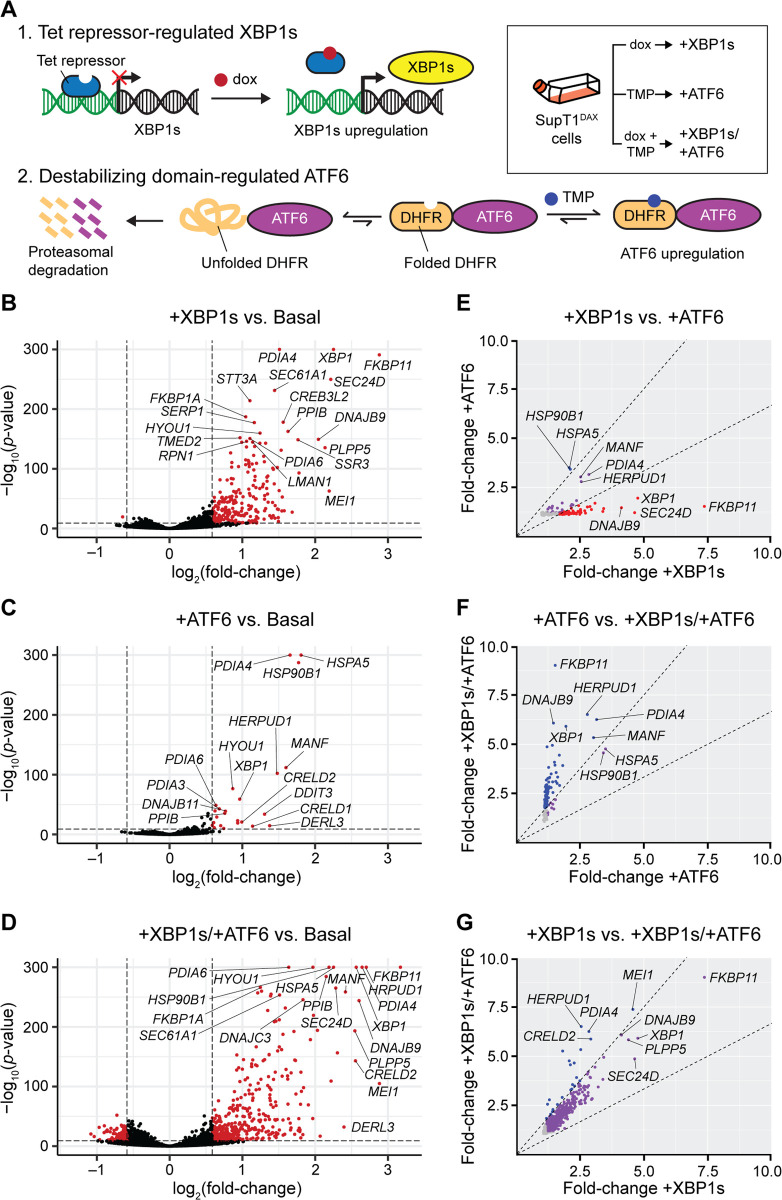
Stress-independent induction of XBP1s, ATF6, or XBP1s and ATF6 creates 4 distinct endoplasmic reticulum proteostasis environments in SupT1^DAX^ cells (basal, +XBP1s, +ATF6, and +XBP1s/+ATF6). (A) Chemical genetic strategy to orthogonally regulate XBP1s and ATF6 in SupT1^DAX^ cells. (B–D) RNA sequencing (RNA-Seq) analysis of the transcriptomic consequences of (B) XBP1s, (C) ATF6, and (D) XBP1s/ATF6 induction. Transcripts that were differentially expressed under each condition based on a >1.5-fold change in expression level (for dox-, TMP-, or dox- and TMP-treated versus vehicle-treated cells) and a non-adjusted *p*-value < 10^−10^ are separated by dashed lines and plotted in red, with select transcripts labeled. The lowest nonzero *p*-value recorded was 10^−291^; therefore, *p*-values equal to 0 were replaced with *p*-value = 1.00 × 10^−300^ for plotting purposes. Transcripts for which *p*-values could not be calculated owing to extremely low expression or noisy count distributions were excluded from plotting. (E–G) Comparison of transcript fold change upon (E) +XBP1s versus +ATF6, (F) +ATF6 versus +XBP1s/+ATF6, and (G) +XBP1s versus +XBP1s/+ATF6 remodeling of the endoplasmic reticulum proteostasis network. Only transcripts with false-discovery-rate-adjusted *p*-value < 0.05 and fold increase > 1 in both of the indicated conditions are plotted. Dashed lines indicate a 1.5-fold filter to assign genes as selectively induced by the proteostasis condition on the *x*-axis (red), *y*-axis (blue), or lacking selectivity (purple). Transcripts with fold increase < 1.2 in either proteostasis environment are colored in grey to indicate low differential expression. The complete RNA-Seq differential expression analysis is provided in S1 Data. dox, doxycycline; TMP, trimethoprim.

To allow for robust replication of HIV, we chose human T cell lymphoblasts (SupT1 cells) as the host cells. SupT1 cells support high levels of HIV replication in cell culture, likely due to the lack of cytidine deaminase activity that can cause hypermutation of HIV DNA [44]. Moreover, infection with HIVeGFP/VSV-G virus or HIV itself does not alter the expression of UPR-controlled genes in SupT1 cells [45,46]. To attain user control of the IRE1-XBP1s and ATF6 transcriptional response in these cells, we used a previously described method of stable cell line engineering [41] (detailed in Materials and Methods). Briefly, the XBP1s transcription factor was placed under control of the tetracycline receptor, and induced by treatment with doxycycline (dox). Orthogonally, the active form of the ATF6 transcription factor was fused to an *Escherichia coli* dihydrofolate reductase (DHFR)–based destabilizing domain, and induced by treatment with trimethoprim (TMP). We termed the resulting engineered cells SupT1^DAX^ cells (Fig 1A), with the DAX signifier indicating the inclusion of both the DHFR.ATF6 and XBP1s constructs.

With stably engineered SupT1^DAX^ cells in hand, we anticipated that we could create 4 distinct ER proteostasis environments (basal, XBP1s-induced, ATF6-induced, and XBP1s/ATF6 co-induced) to assess potential consequences for Env mutational tolerance. We induced the XBP1s and ATF6 transcriptional responses in SupT1^DAX^ cells, either separately or together, and evaluated resultant changes in the transcriptome using RNA sequencing (RNA-Seq) (S1 Data). We applied gene set enrichment analysis [47] to the RNA-Seq results using the MSigDB C5 collection, and found that gene sets related to ER stress, Golgi trafficking, and ERAD were highly enriched upon induction of XBP1s, induction of ATF6, and co-induction of XBP1s and ATF6 (S2 Data). In contrast, gene sets that serve as markers of other stress responses (e.g., the heat shock response) were not enriched, consistent with a highly selective, stress-independent induction of UPR transcriptional responses.

Comparing the resulting transcriptomes, we observed significant and substantial upregulation of 223 transcripts upon XBP1s induction (+XBP1s), 24 transcripts upon ATF6 induction (+ATF6), and 436 transcripts upon co-induction of XBP1s and ATF6 (+XBP1s/+ATF6) (Fig 1B–1D). For all 3 treatment conditions, the upregulated transcripts were strongly biased towards known UPR-regulated components of the ER proteostasis network.

To analyze the extent to which these 3 perturbations (+XBP1s, +ATF6, and +XBP1s/+ATF6) engendered unique ER proteostasis environments, we cross-compared the mRNA fold changes owing to each treatment (Fig 1E–1G). Transcripts known to be targeted primarily by XBP1s were strongly upregulated upon dox treatment (e.g., *SEC24D* and *DNAJB9*), whereas transcripts known to be targeted primarily by ATF6 were more strongly upregulated upon TMP treatment (e.g., *HSP90B1* and *HSPA5*) (Fig 1E) [41,48,49]. We used immunoblotting to confirm successful induction of these pathways, observing selective protein-level induction of the XBP1s target Sec24D upon dox treatment versus selective induction of the ATF6 target BiP (*HSPA5*) upon TMP treatment (S1 Fig). XBP1s induction caused an extensive remodeling of the entire ER proteostasis network, whereas ATF6 induction resulted in targeted upregulation of just a select subset of ER proteostasis factors, consistent with prior work showing that ATF6 induction causes upregulation of fewer transcripts than XBP1s [41,49]. Notably, the combined induction of XBP1s and ATF6 provided access to a third environment where specific transcripts (e.g., genes known to be targets of XBP1s and ATF6 heterodimers, such as *HERPUD1*) were more strongly upregulated than upon the single induction of either transcription factor (Fig 1F and 1G) [41,50,51]. Taken together, our RNA-Seq results show that we can access 4 distinctive ER proteostasis environments for Env mutational tolerance experiments via chemical genetical control of XBP1s and ATF6 (basal, +XBP1s, +ATF6, and +XBP1s/+ATF6).

We assessed whether these perturbations of the ER proteostasis environment had deleterious effects on cell viability or restricted HIV replication, as we had previously observed inhibition of HIV replication upon upregulation of the heat shock response [52]. To address the former, we induced XBP1s and ATF6, individually or simultaneously, in SupT1^DAX^ cells and measured resazurin metabolism 72 h after drug treatment (S2A Fig). We observed that the perturbed proteostasis conditions did not alter the metabolic activity of SupT1^DAX^ cells, consistent with no deleterious effects on cell viability. To address whether HIV replication was restricted, we used the TZM-bl assay to quantify HIV infectious titer (S2B Fig). Specifically, we used TZM-bl reporter cells containing the *E*. *coli* β-galactosidase gene under the control of an HIV long terminal repeat sequence [53]. When these cells are infected with HIV, the HIV Tat transactivation protein induces expression of β-galactosidase, which cleaves the chromogenic substrate (X-Gal) and causes infected cells to appear blue in color. The infectious titer increased marginally by approximately 3.5-fold when XBP1s was induced, either alone or together with ATF6. Induction of ATF6 alone did not affect HIV infectious titer. Thus, ER proteostasis network perturbation via XBP1s and/or ATF6 induction did not deleteriously impact HIV replication.

### Env DMS in 4 distinct host ER proteostasis environments

We next applied DMS to Env to test our hypothesis that the composition of the host’s ER proteostasis network plays a central role in determining the mutational tolerance of Env. For this purpose, we employed a previously developed set of 3 replicate Env proviral plasmid libraries [22], created by introducing random codon mutations at amino acid residues 31–702 of the Env protein (note that the HXB2 numbering scheme [54] is used throughout). Briefly, the library was generated using a previously described technique that uses pools of primers containing a random NNN nucleotide sequence at the codon of interest, and introduces mutations via iterative rounds of low-cycle PCR [55]. This technique generates multi-nucleotide (e.g., gca → gAT) as well as single nucleotide (e.g., gca → gAa) codon mutations, thereby introducing mutations at the codon level rather than at the nucleotide level [22,55]. The N-terminal signal peptide and the C-terminal cytoplasmic tail of Env were excluded from mutagenesis owing to their dramatic impact on Env expression and/or HIV infectivity [22].

**Fig 2:**
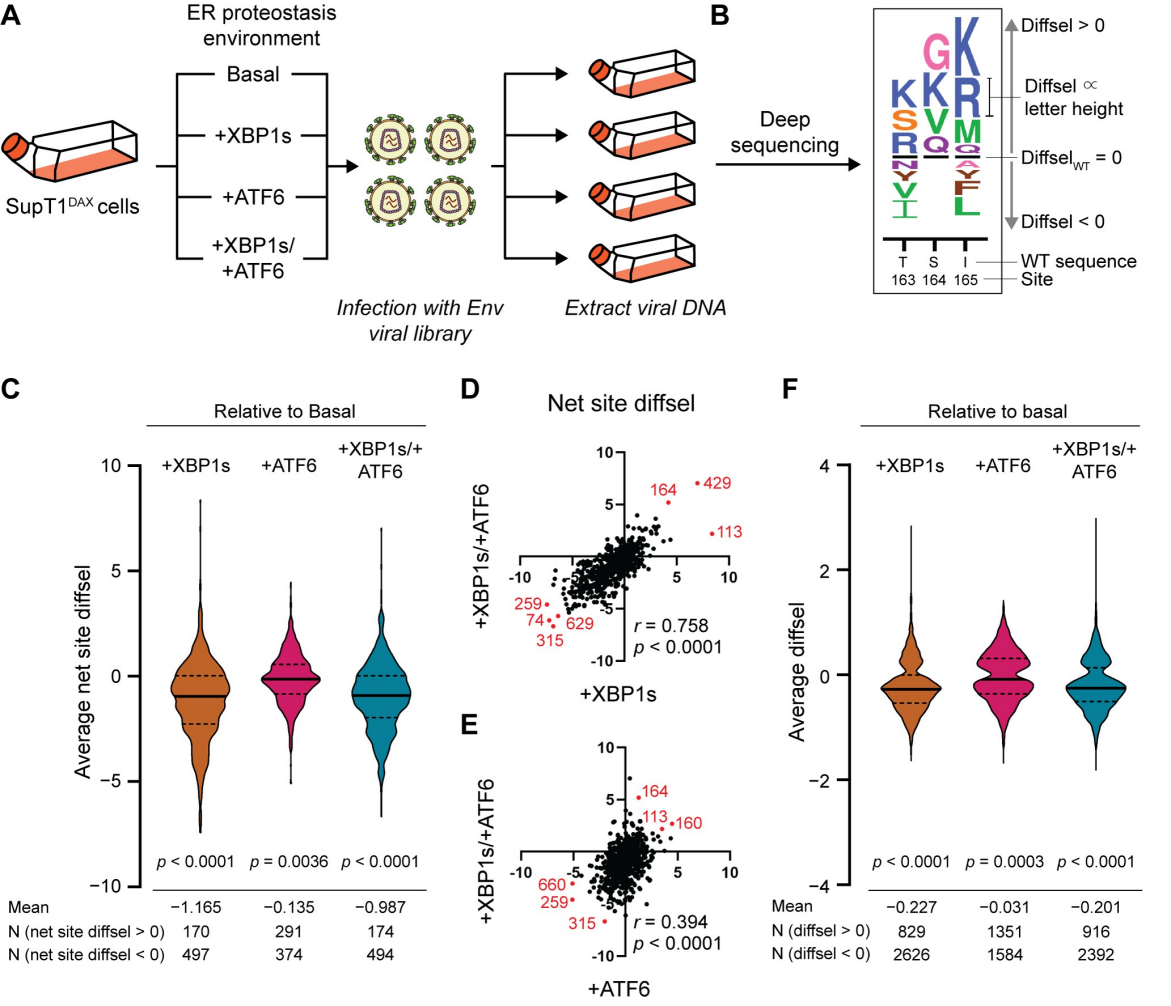
Upregulation of the host cell’s ER proteostasis environment generally reduces mutational tolerance across the Env protein sequence. (A) Scheme for deep mutational scanning of Env in 4 distinct ER proteostasis environments (basal, +XBP1s, +ATF6, and +XBP1s/+ATF6). SupT1^DAX^ cells were pretreated with DMSO (basal), dox (+XBP1s), TMP (+ATF6), or both dox and TMP (+XBP1s/+ATF6) 18 h prior to infection with biological triplicate Env viral libraries. 4 d post-infection, cells were harvested, and non-integrated viral DNA was sequenced to quantify the diffsel of Env variants. (B) Diffsel for each amino acid variant can be visualized in a sequence logo plot. The black horizontal lines at the center represent the diffsel for the wild-type amino acid at that site, and the height of the amino acid letter abbreviations is proportional to the diffsel of that variant in the remodeled ER proteostasis environment relative to the basal environment. Variants that are relatively enriched in the indicated ER proteostasis environment (positive diffsel) are located above the black horizontal line. Variants that are relatively depleted in the indicated ER proteostasis environment (negative diffsel) are located below the black horizontal line. (C) Net site diffsel for all Env sites in 3 perturbed ER proteostasis environments, averaged over biological triplicates. The black horizontal lines on the violin plots indicate the median (solid line) and the first and the third quartiles (dashed lines) of the distribution. The significance of deviation from null (net site diffsel = 0, no selection) was tested using a 1-sample *t* test, with 2-tailed *p*-values shown. The mean of the distribution and the number of sites with net site diffsel >0 or <0 are listed below the distribution. (D and E) Correlation for net site diffsel values for (D) +XBP1s/+ATF6 versus +XBP1s and (E) +XBP1s/+ATF6 versus +ATF6, normalized to the basal proteostasis environment. Pearson correlation coefficients (*r*) and corresponding *p*-values are shown. Select sites with highly positive or highly negative net site diffsel values in both proteostasis environments are marked in red and labeled with site numbers. (F) Diffsel for individual Env variants in 3 perturbed ER proteostasis environments, averaged over biological triplicates. The black horizontal lines on the violin plots indicate the median (solid line) and the first and the third quartiles (dashed lines) of the distribution. The significance of deviation from null (diffsel = 0, no selection) was tested using a 1-sample *t* test, with 2-tailed *p*-values shown. The mean of the distribution and the number of sites with diffsel >0 and <0 are listed below the distribution. Diffsel values (C–F) are provided at https://github.com/yoon-jimin/2021_HIV_Env_DMS. diffsel, differential selection; dox, doxycycline; ER, endoplasmic reticulum; TMP, trimethoprim; WT, wild-type.

We generated biological triplicate viral libraries from these mutant Env plasmid libraries by transfecting the plasmid libraries into HEK293T cells and then harvesting the passage 0 (p0) viral supernatant after 4 d. Deep sequencing of the 3 p0 viral libraries showed that 74% of all possible amino acid substitutions were observed at least 3 times in each of the triplicate libraries, and 98% of all possible substitutions were observed at least 3 times in at least 1 of the triplicate libraries, consistent with prior work [22,36]. Mutations that were not included in the viral libraries were dispersed throughout the sequence and did not correspond to specific regions of structural or functional importance (S3 Fig). To establish a genotype–phenotype link, we passaged the p0 transfection supernatants in SupT1 cells at a very low multiplicity of infection (MOI) of 0.005 infectious virions/cell. We next performed batch competitions of each individual Env viral library in SupT1^DAX^ cells in each of the 4 different ER proteostasis environments: basal, +XBP1s, +ATF6, and +XBP1s/+ATF6 (Fig 2A). Briefly, SupT1^DAX^ cells were treated with vehicle, dox, TMP, or both dox and TMP to generate the intended ER proteostasis environment, followed by infection with p1 viral supernatant at a MOI of 0.005 infectious virions/cell. We used this MOI to minimize co-infection of individual cells and thereby maintain the genotype–phenotype link. Non-integrated viral DNA was extracted, and *Env* amplicons were generated by PCR [22]. Finally, we deep-sequenced the amplicons using barcoded-subamplicon sequencing (S4 Fig) and analyzed the sequencing reads using the dms_tools2 suite (https://jbloomlab.github.io/dms_tools2/) [56,57].

To identify amino acid variants that were differentially enriched or depleted in a given ER proteostasis selection condition (+XBP1s, +ATF6, or +XBP1s/+ATF6) relative to the basal ER proteostasis environment, we quantified differential selection (diffsel) (Fig 2B). Diffsel was calculated by taking the logarithm of the variant’s enrichment in the selection condition relative to its enrichment in the basal ER proteostasis network condition [57]. For example, if a variant exhibited positive diffsel in +XBP1s (selection) versus basal (mock), it would indicate that the variant was more enriched relative to the wild-type amino acid in the +XBP1s condition compared to the basal condition. In addition, to decipher reliable signal from experimental noise, we filtered the DMS data using a previously described and validated 2-step strategy [32]. First, we removed variants that were not present in all 3 pre-selection replicate viral libraries. That is, we eliminated even those variants that were strongly enriched or depleted in 2 replicates if they were not present in the starting library of the third replicate. Second, we removed variants that exhibited diffsel in opposite directions in any of the biological triplicates. Using the second filter, we typically removed variants that were minimally affected by the selection, displaying slightly positive diffsel values in one replicate but slightly negative diffsel values in another. By applying these 2 filters, we were able to focus subsequent analyses only on Env variants that exhibited robust, reproducible diffsel across biological triplicates of the same ER proteostasis network conditions (out of 12,787 theoretically possible non-wild-type variants: 3,455 variants for +XBP1s [27%], 2,935 variants for +ATF6 [23%], and 3,308 variants for +XBP1s/+ATF6 [26%]).

### XBP1s induction causes a strong net decrease in the mutational tolerance of Env, consistent with enhanced quality control of biophysically defective variants

To evaluate our hypothesis that the composition of the host’s ER proteostasis network critically shapes Env mutational tolerance, we first analyzed the “net site diffsel” in each host ER proteostasis environment. Net site diffsel is the sum of individual mutational diffsel values for a given Env site. Thus, a positive net site diffsel indicates that mutational tolerance at a given Env site is quantitatively increased in the enhanced host ER proteostasis environment relative to the basal ER proteostasis environment. In contrast, a negative net site diffsel indicates that mutational tolerance is decreased in the enhanced host ER proteostasis environment. For example, the net site diffsel for site 164 (Fig 2B) would be the sum of the diffsel values for G, K, V, and Q, which would be positive; therefore, we would conclude that the overall mutational tolerance, as defined here, increased at site 164.

Using the filtered Env DMS datasets, we calculated net site diffsel at each Env position averaged across the 3 biological replicates of our experiment (Fig 2C). Strikingly, the +XBP1s ER proteostasis environment globally, substantially, and significantly reduced mutational tolerance across the entire Env protein (mean net site diffsel = −1.165, *p*-value < 0.0001). Co-induction of XBP1s and ATF6 had a similar effect, again substantially and significantly reducing Env mutational tolerance (mean net site diffsel = −0.987, *p*-value < 0.0001). The magnitude of absolute mean net site diffsel was approximately 14-fold larger upon XBP1s induction than we previously observed for increased mutational tolerance in influenza hemagglutinin in an XBP1s-activated ER proteostasis environment at 37°C [32]. Thus, Env mutational tolerance is exceptionally sensitive to XBP1s-mediated ER proteostasis network upregulation, to a much greater extent than hemagglutinin. In contrast, the +ATF6 ER proteostasis environment, while still mildly reducing mutational tolerance across Env, had a less substantial global effect (mean net site diffsel = −0.135, *p*-value = 0.0036). The latter result suggests that the reduced Env mutational tolerance observed in the +XBP1s/+ATF6 ER proteostasis environment was largely driven by ER proteostasis factors targeted by XBP1s. Indeed, the Pearson correlation coefficient *r* was substantially higher between the net site diffsel values observed in the +XBP1s versus +XBP1s/+ATF6 environments (*r* = 0.758; Fig 2D) than between those observed in the +ATF6 versus +XBP1s/+ATF6 environments (*r* = 0.394; Fig 2E). This observation aligns well with our RNA-Seq data, in which we observed substantially more overlap between the ER proteostasis network transcriptome remodeling caused by XBP1s induction and that caused by the co-induction of XBP1s and ATF6, than between that caused by ATF6 induction and that caused by co-induction (Fig 1F and 1G).

It is important to note that, in a net site diffsel analysis, we quantify the relative enrichment of all amino acid variants combined to assess mutational tolerance at a given Env site. Consequently, a decrease in mutational tolerance as measured by net site diffsel could be caused by a single amino acid variant that was strongly disfavored or, alternatively, by many variants being disfavored relative to wild type. To test if individual amino acid variants also reveal a global tendency towards reduced mutational fitness, we plotted the individual diffsel values for all Env variants. We again observed reduced mutational fitness of the majority of Env variants whenever XBP1s was induced, indicating that the effect is largely driven by a general loss of mutational tolerance rather than by just a few specific amino acid variants being strongly disfavored (Fig 2F).

**Fig 3:**
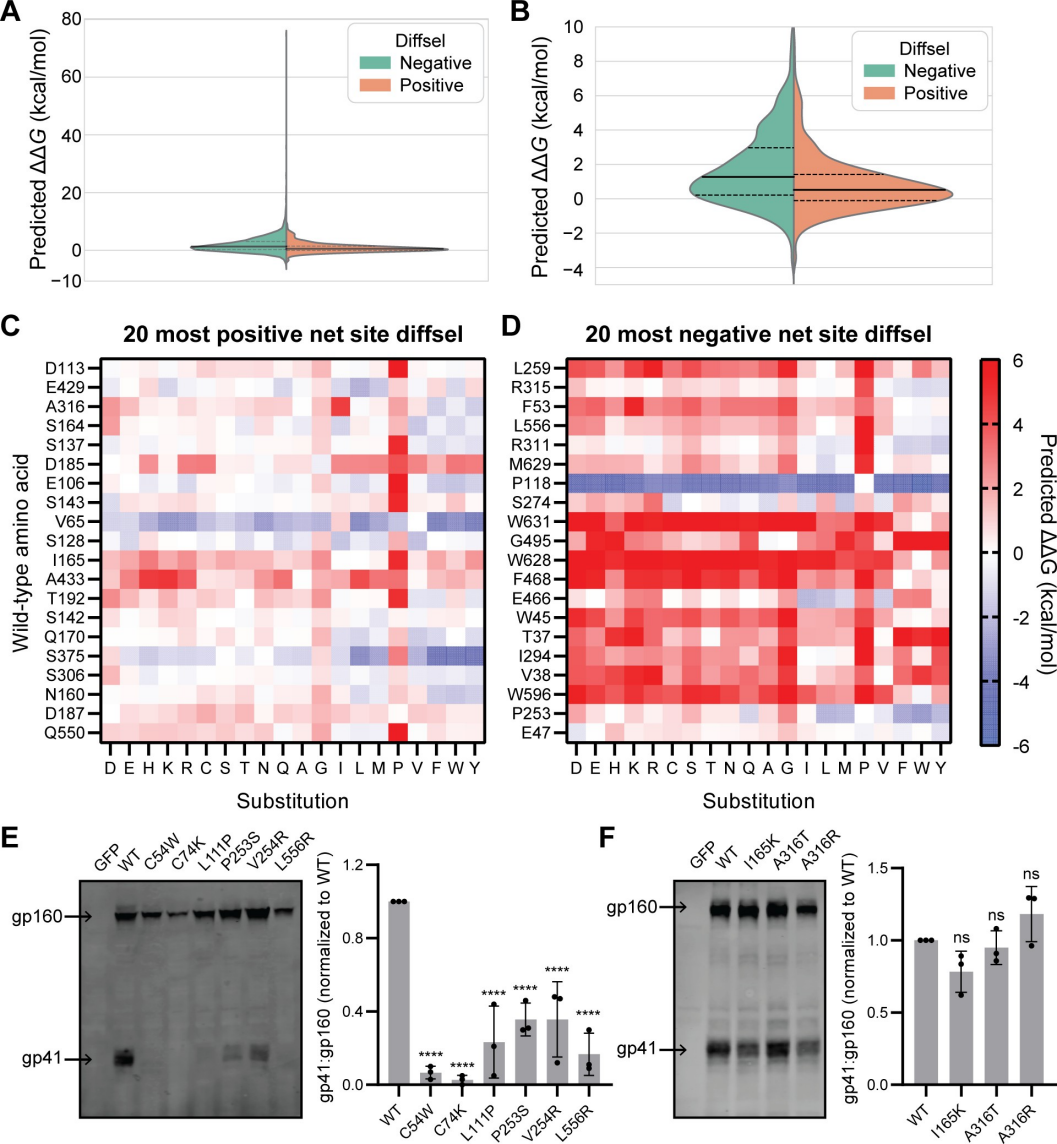
Env variants displaying negative differential selection (diffsel) upon XBP1s induction tend to be more destabilizing and exhibit greater processing defects than those displaying positive diffsel. (A) Split violin plot depicting the distribution of ΔΔ*G* values predicted using the Rosetta ΔΔ*G* protocol, for all amino acid substitutions that were present in the filtered deep mutational scanning dataset for +XBP1s versus basal (2,379 negative diffsel variants; 756 positive diffsel variants). Dashed lines inside the violins indicate the first and third quartiles, and the solid line inside the violins indicates the median. (B) Zoom-in of the violin plot in (A) focusing on ΔΔ*G* < 10 kcal/mol. (C and D) Heatmaps showing the predicted ΔΔ*G* values for all possible amino acid substitutions at the 20 sites with the most positive net site diffsel (C) and the 20 sites with the most negative net site diffsel (D), upon XBP1s induction. Substitutions (*x*-axis) are arranged by side-chain properties: negatively charged (D, E), positively charged (H, K, R), polar uncharged (C, S, T, N, Q), small nonpolar (A, G), aliphatic (I, L, M, P, V), and aromatic (F, W, Y). Wild-type (WT) amino acids (*y*-axis) are arranged by rank order of net site diffsel, with (C) D113 most positive and (D) L259 most negative. Complete ΔΔ*G* values are provided in S5 Data. (E) Representative immunoblot showing gp160 and gp41 bands for selected variants with negative diffsel upon XBP1s induction (left) and densitometric analysis of gp41:gp160 ratio across biological triplicates (right). (F) Representative immunoblot showing gp160 and gp41 bands for selected variants with positive diffsel upon XBP1s induction (left) and densitometric analysis of the gp41:gp160 ratio across biological triplicates (right). For (E) and (F), statistical significance was calculated by 1-way ANOVA followed by Dunnett’s test, comparing the mean of each variant to the mean of WT; ****p-value < 0.0001; ns, not significant. Immunoblots of biological triplicates are provided in S5 Fig, and replicate data values for densitometric analysis are provided in S6 Data.

The unanticipated and striking decrease in mutational tolerance of Env upon XBP1s induction could potentially arise from the fact that XBP1s upregulates both chaperones that assist client protein folding and quality control factors that identify and dispose of defective proteins. We used the Rosetta ΔΔ*G* protocol to predict the energetic consequences of all amino-acid substitutions that were present in our filtered DMS dataset (S5 Data) [58]. Although there are limitations associated with using the Rosetta cartesian_ddg protocol to predict exact, absolute changes in protein folding free energy upon substitution, the protocol and associated scaling factors can provide the relative stability of substitutions and a general classification between destabilizing and stabilizing substitutions [58]. Disulfide-bonding cysteine residues, which the Rosetta protocol defines as a feature and for which it disallows substitutions, were excluded from ΔΔ*G* prediction, although substitutions in these disulfide-bonding cysteines can be presumed to be highly destabilizing owing to the critical structural roles of disulfide bonds. To test whether the variants that exhibit negative diffsel values upon XBP1s induction are more destabilizing than those with positive diffsel values, we compared the distribution of predicted ΔΔ*G* for all variants with positive diffsel versus negative diffsel (Fig 3A and 3B). We observed that the variants with negative diffsel on average had moderately higher (more destabilizing) predicted ΔΔ*G* than the variants with positive diffsel (2-sample *t* test, 2-tailed *p*-value < 0.0001). To further test if substitutions at mutationally intolerant sites upon XBP1s induction are generally destabilizing, we focused on the 20 most negative and the 20 most positive net site diffsel positions (Fig 3C and 3D). We again found that, overall, substitutions at sites with strongly negative net site diffsel (sites with low mutational tolerance) were much more destabilizing than substitutions at sites with strongly positive net site diffsel (sites with high mutational tolerance).

Rosetta ΔΔ*G* only makes predictions regarding thermodynamic stability, whereas variants can also induce defects in the kinetics of folding or proper processing of Env. We next used an experimental approach to assess whether variants displaying negative diffsel values upon XBP1s induction displayed more serious trafficking defects than those with positive diffsel values. Since Env is synthesized as a precursor protein (gp160) in the ER and proteolytically cleaved into gp120 and gp41 in the Golgi apparatus, Env variants that fail to pass ER quality control would be predicted to result in a lower gp41:gp160 or gp120:gp160 ratio compared to wild-type Env [59–61]. We chose 6 variants with strongly negative diffsel (C54W, C74K, L111P, P253S, V254R, and L556R) and 3 variants with strongly positive diffsel (I165K, A316T, and A316R) upon XBP1s induction, transfected them into HEK293T cells, and determined the steady-state ratio of gp41 to gp160 using immunoblotting. We observed that all the tested Env variants with negative diffsel exhibited lower gp41:gp160 than wild-type Env (Fig 3E), while the ratio was only slightly lower or sometimes higher than wild-type Env for variants with positive diffsel (Fig 3F). Of note, gp41 bands were nearly undetectable when substitutions were made at disulfide-bonding cysteines, confirming that substitutions at these cysteines do severely disrupt Env trafficking. Together, our Rosetta ΔΔ*G* predictions and experimental data strongly support the hypothesis that the Env variants rendered less fit upon XBP1s induction were more energetically destabilizing and disrupted Env maturation more strongly than the variants that were enriched upon XBP1s induction.

While it is known that infection with HIVeGFP/VSV-G virus or HIV itself does not result in UPR upregulation in SupT1 cells [45,46], it is possible that the destabilized or poorly folding variants in our library may significantly misfold in the ER and result in more pronounced UPR activation. To address this possibility, we transfected wild-type Env and Env variants that were strongly negatively selected (C54W, L111P, and L556R) into HEK293T cells (instead of SupT1 cells, where high-efficiency transfection is not possible) and measured UPR upregulation using real-time PCR (S6 Fig). Overall, both the wild-type Env and the 3 variants displayed UPR signaling equivalent to GFP-transfected cells (negative control), and to a much lower level than the GFP-transfected cells treated with the ER stress inducer thapsigargin (positive control). This result indicates that it is unlikely that the destabilized variants in our library activated the UPR above the basal level.

In sum, there is a striking decrease in mutational tolerance across much of Env upon XBP1s-mediated remodeling of the host’s ER proteostasis network. Although unexpected, this observation is actually quite consistent with XBP1s-upregulated quality control factors restricting the available protein sequence space by enacting stringent quality control on biophysically defective protein variants. This broad and substantive tendency should not, however, mask the fact that numerous sites displayed strongly enhanced mutational tolerance upon not just XBP1s induction but also ATF6 induction (e.g., S164 and D113) (Fig 2C–2E). Finally, it should be noted that although ATF6 induction had minimal global consequences for Env mutational tolerance, there were still a number of sites where reduced net site diffsel (e.g., L259 and R315) was observed across all 3 enhanced ER proteostasis environments (Fig 2D and 2E).

### Investigation of Env sites and variants most strongly impacted by the host’s ER proteostasis network

**Fig 4:**
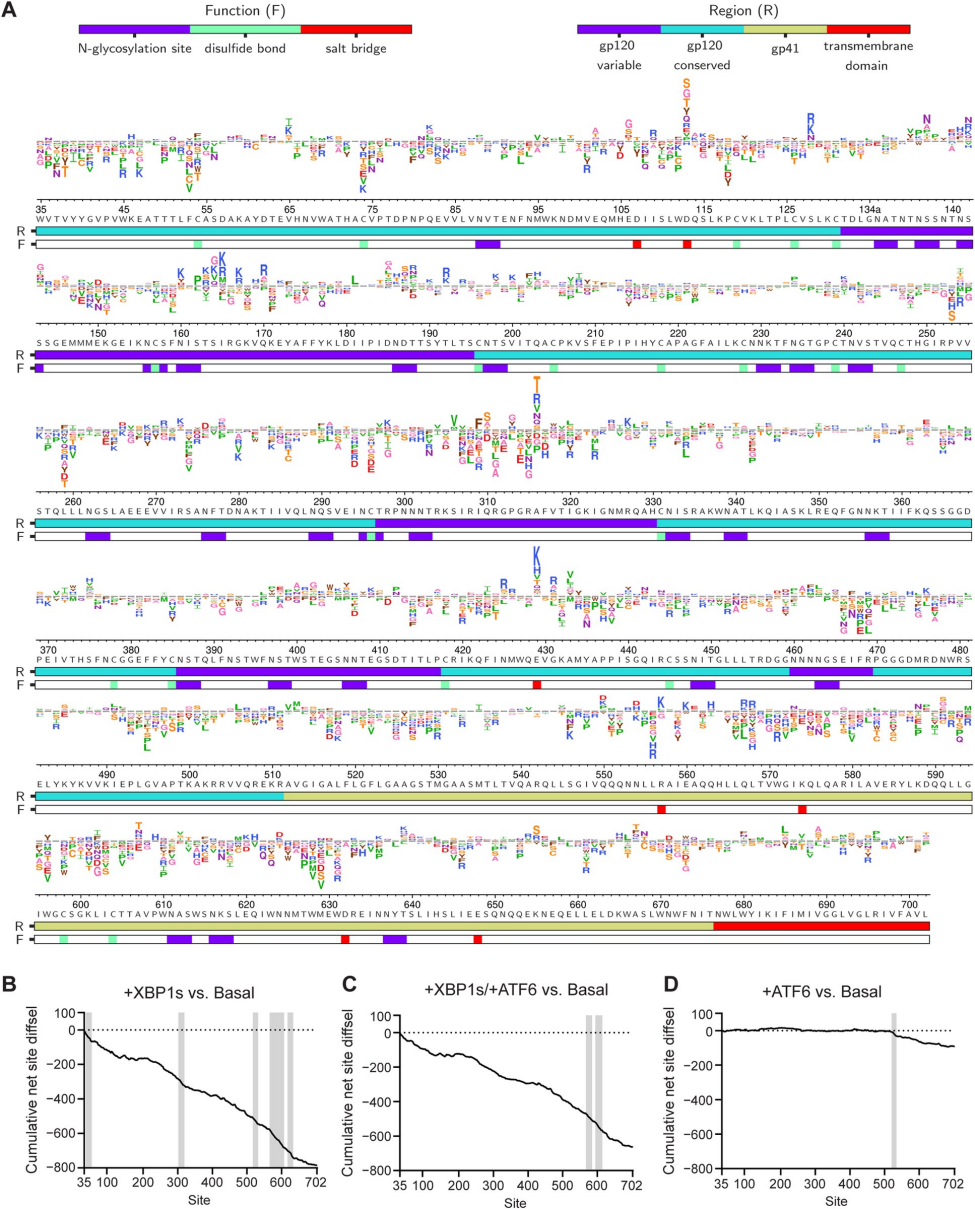
Differential selection (diffsel) across Env upon remodeling of the host’s endoplasmic reticulum proteostasis network. (A) Logo plot displaying averaged diffsel for +XBP1s normalized to the basal proteostasis environment. The height of the amino acid abbreviation is proportional to the magnitude of diffsel. The amino acid abbreviations are colored based on their side-chain properties: negatively charged (D, E; red), positively charged (H, K R; blue), polar uncharged (C, S, T; orange/N, Q; purple), small nonpolar (A, G; pink), aliphatic (I, L, M, P, V; green), and aromatic (F, W, Y; brown). The numbers and letters below the logos indicate the Env site in HXB2 numbering and the identity of the wild-type amino acid for that site, respectively. The color bar below the logos indicates the function (F) that the site is involved in (*N*-glycosylation site [purple], disulfide bond [green], or salt bridge [red]) or the region (R) of Env that the site belongs to (gp120–variable [purple], gp120–conserved [cyan], gp41 [yellow], or transmembrane domain [red]; the sites that belong to the 5 variable loops of gp120 were categorized as “gp120–variable,” and the sites that are not included in the 5 variable loops were categorized as “gp120–conserved”). Only variants that were present in all 3 pre-selection viral libraries and exhibited diffsel in the same direction across all 3 biological triplicates are plotted here. Diffsel values and unfiltered logo plots for each individual replicate are provided at https://github.com/yoon-jimin/2021_HIV_Env_DMS. (B–D) Cumulative net site diffsel across Env sites for (B) +XBP1s, (C) +XBP1s/+ATF6, and (D) +ATF6, normalized to the basal proteostasis environment. Regions where the decrease in mutational tolerance is particularly prominent are shaded in grey (40–57, 302–319, 517–532, 565–607, and 617–633 for [B], 567–585 and 594–614 for [C], and 520–534 for [D]). Cumulative net site diffsel data values are provided in S8 Data.

To visualize the relative fitness of individual amino acid variants in each host ER proteostasis environment, we generated sequence logo plots across the entire Env sequence (Figs 4A, S7, and S8). The relative enrichment for each amino acid variant (diffsel) was calculated from our filtered datasets by averaging across 3 biological replicates. The unfiltered, unaveraged full sequence logo plots for each replicate and condition are also provided at https://github.com/yoon-jimin/2021_HIV_Env_DMS.

Several features of these logo plots were immediately noteworthy. First, the global and relatively similar reduction in mutational tolerance caused by XBP1s induction (Fig 4A) and co-induction of XBP1s and ATF6 (S7 Fig) was readily observed. To visualize this phenomenon and highlight specific regions in which the effect size is particularly large, we plotted cumulative net site diffsel against Env sites (Fig 4B–4D). We observed that the decrease in mutational tolerance was most prominent around the following sites: 40–57, 302–319, 517–532, 565–607, and 617–633 when XBP1s was induced alone (Fig 4A and 4B); 567–585 and 594–614 when XBP1s and ATF6 were co-induced (Figs 4C and S7), and 520–534 when ATF6 was induced alone (Figs 4D and S8), as indicated by the steeper slopes in those regions. In all 3 proteostasis environments, sites with strong decreases in mutational tolerance included regions in gp41 (residues 512–702). Second, although the general tendency towards reduced mutational tolerance was quite striking, it was also apparent that there are specific positions where either XBP1s- or ATF6-mediated ER proteostasis network enhancement strongly enhanced mutational tolerance at a given site (e.g., D113) or enhanced the fitness of a specific variant (e.g., I309F). We assessed whether this differential impact of ER proteostasis mechanisms was related to the surface accessibility of sites, but did not observe a strong linear correlation between net site diffsel and surface accessibility across Env sites for either the Env monomer or the trimer (S9 Fig). Still, we observed that when XBP1s was induced, either alone or together with ATF6, sites that had high surface accessibility were more likely to have positive net site diffsel than sites that had low surface accessibility. Third, the stronger impacts of XBP1s induction compared to ATF6 induction were apparent (Fig 4A versus S8 Fig, and Fig 4B versus 4D).

**Fig 5:**
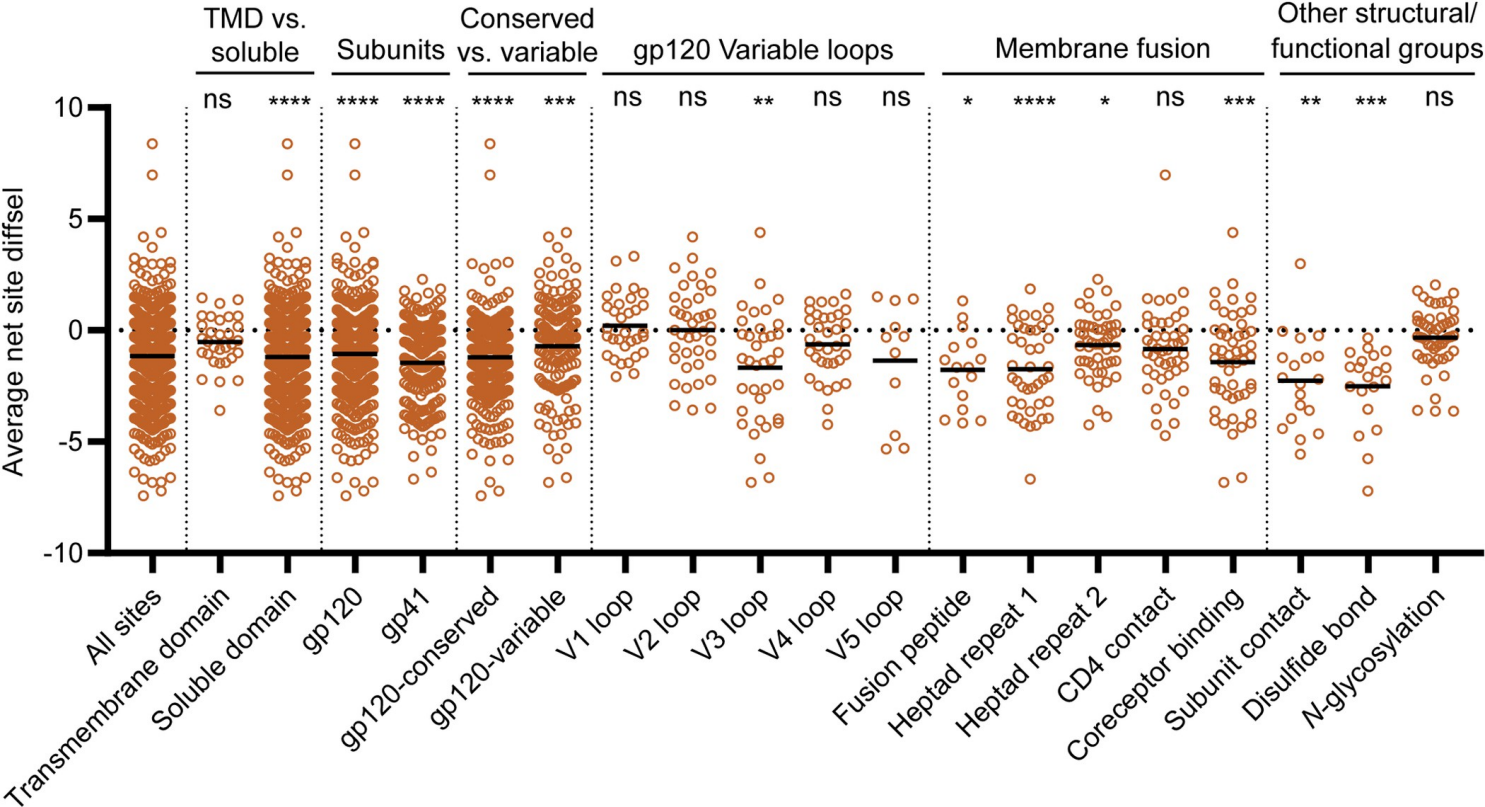
Impact of XBP1s induction on mutational tolerance varies across Env structural elements. Average net site differential selection (diffsel) for the +XBP1s endoplasmic reticulum (ER) proteostasis environment normalized to the basal ER proteostasis environment, where the means of the distributions are indicated by black horizontal lines. Sites are sorted by transmembrane domain (TMD) versus soluble, subunits, conserved versus variable regions of gp120, the 5 variable loops of gp120, regions important for membrane fusion, and other structural/functional groups. For TMD versus soluble, all sites that do not belong to the TMD were categorized as “soluble.” For conserved versus variable, the sites that belong to the 5 variable loops of gp120 were categorized as “gp120–variable,” and the sites that are not included in the 5 variable loops were categorized as “gp120–conserved.” Significance of deviation from null (net site diffsel = 0, no selection) was tested using a 1-sample *t* test. The derived *p*-values were Bonferroni-corrected for 20 tests; *p-value < 0.05, **p-value < 0.01, ***p-value < 0.001, ****p-value < 0.0001; ns, not significant. Diffsel values are provided at https://github.com/yoon-jimin/2021_HIV_Env_DMS. Assignment of structural regions is provided in S2 Table.

To assess whether or not the global decrease in mutational tolerance could be attributed to specific structural or functional regions, we calculated the average net site diffsel for individual functional/structural groups. These groups included (1) the transmembrane and soluble domains; (2) the entire gp120 and gp41 subunits; (3) the conserved and variable regions of gp120, where the conserved region is defined as the region that does not belong to the 5 variable loops of gp120; (4) the 5 variable loops of gp120 individually (denoted V1–V5); (5) regions responsible for viral membrane fusion; and (6) other sites with important functional and structural roles (Figs 5, S10, and S11; see corresponding references for assignment of these regions in S2 Table).

We focused first on the consequences of XBP1s induction because the effects were larger than for ATF6 induction and similar to the consequences of co-induction. We examined the mutational tolerance of the transmembrane domain (TMD) of Env, since recent studies have suggested that the TMDs of other membrane proteins exhibit particularly restricted mutational tolerance (Fig 5, “TMD vs. soluble”) [18,62]. We observed a reduction in mutational tolerance for the TMD, but the difference was not statistically significant, and the mean net site diffsel for the TMD was less negative than that of the soluble domains. While it is certainly possible that the TMD of Env has highly restricted mutational tolerance, that mutational tolerance (or intolerance) was not particularly altered by XBP1s induction.

**Fig 6:**
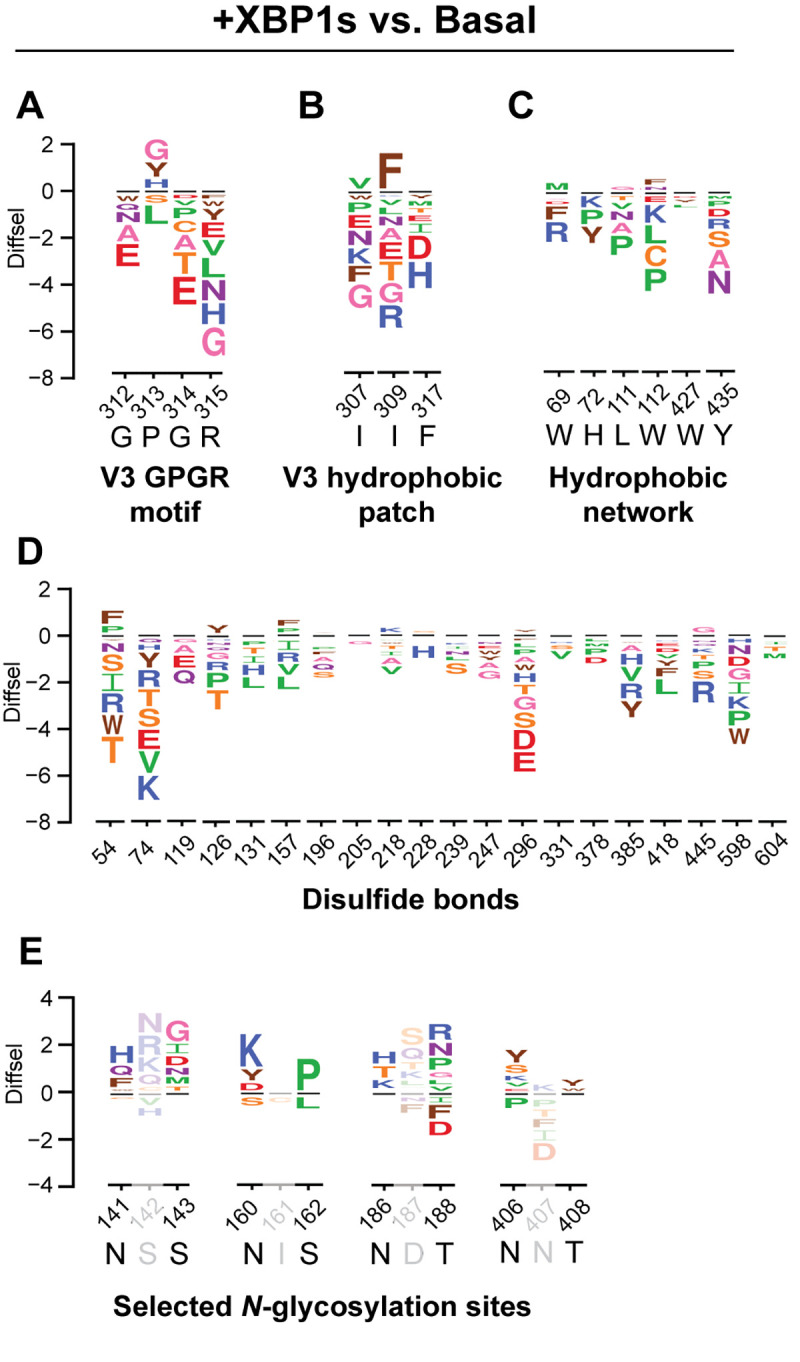
Diverse functional elements of Env respond differently to XBP1s induction. Selected sequence logo plots for the +XBP1s endoplasmic reticulum (ER) proteostasis environment normalized to the basal ER proteostasis environment for (A) the conserved GPGR motif of the V3 loop, (B) the hydrophobic patch of the V3 loop, (C) the hydrophobic network of gp120 (important for CD4 binding), (D) cysteine residues participating in disulfide bonds, and (E) selected *N*-glycosylation sequons (N-X-S/T) that exhibited positive net site differential selection (diffsel) in all 3 remodeled proteostasis environments. The height of the amino acid abbreviation corresponds to the magnitude of diffsel. The numbers and letters below the logos indicate the Env site in HXB2 numbering and the wild-type amino acid for that site, respectively. Only variants that were present in all 3 pre-selection viral libraries and exhibited diffsel in the same direction across the biological triplicates are plotted. All logo plots were generated on the same scale. Diffsel values are provided at https://github.com/yoon-jimin/2021_HIV_Env_DMS. Assignments of functional regions are provided in S2 Table.

We observed a decrease in mutational tolerance for both gp120 and gp41, indicating that XBP1s upregulation impacts both subunits of Env, albeit gp41 more strongly (Fig 5, “Subunits”). Within the gp120 subunit, there was a stronger decrease in mutational tolerance for the regions that did not belong to any variable loops (gp120–conserved) than there was for the variable loops (gp120–variable), although both conserved and variable regions exhibited a loss of mutational tolerance (Fig 5, “Conserved vs. variable”). Among the 5 variable loops of gp120, the more conserved V3 loop exhibited the strongest negative net site diffsel (Fig 5, “gp120 Variable loops”) [63]. Further notable within the V3 loop, we observed a particularly large decrease in mutational tolerance for sites that are highly conserved, such as the GPGR motif or the hydrophobic patch whose disruption causes gp120 shedding (Fig 6A and 6B) [64].

To test whether sequence variability correlated with mutational tolerance across the entire Env protein, we plotted net site diffsel against Shannon entropy, which is a measure of sequence variability within Env sequences of various HIV strains. Indeed, although the linear correlation was not high, 53.6% of positions with high Shannon entropy exhibited increases in mutational tolerance, while only 20.5% of positions did so in conserved positions (S12 Fig). These observations suggest that conserved regions in Env generally experience stronger selection pressure when the ER proteostasis network is upregulated than do variable regions.

We next scrutinized Env regions directly involved in membrane fusion, since the principal function of Env in the HIV replication cycle is to facilitate host cell entry via the fusion of viral and host membranes. Briefly, upon binding to cell surface CD4 receptor and coreceptor, the fusion peptide in gp41 is inserted into the cell membrane, and the 2 heptad repeat domains form a 3-stranded coiled coil that allows the anchoring of Env to the host cell membrane [65]. With the exception of CD4 contact sites, regions participating in membrane fusion (Fig 5, “Membrane fusion”) experienced decreased mutational tolerance upon XBP1s induction. In addition, the hydrophobic network of gp120, which undergoes conformational changes upon CD4 binding to trigger membrane fusion [66], exhibited negative net site diffsel (Fig 6C).

Lastly, we focused further attention on regions of Env that may play important roles in Env folding and stability. We observed a significant decrease in mutational tolerance for sites participating in the gp120–gp41 subunit contact (Fig 5, “Subunit contact”). Next, we asked what the consequences of XBP1s induction are for disulfide bonds and *N*-glycosylation sequons. Particularly noteworthy, we observed that every single cysteine residue involved in disulfide bonds exhibited negative net site diffsel upon XBP1s induction (Fig 5, “Disulfide bond”; Fig 6D), consistent with the notion that the XBP1s-remodeled ER proteostasis environment strictly quality-controls disulfide bond formation in Env. The results were different for *N*-glycosylation sequons, even though these residues can also promote ER protein folding and quality control by providing access to the ER’s lectin-based chaperone network [67]. We observed an approximately equal number of sites in *N*-glycosylation sequons that displayed positive and negative net site diffsel upon XBP1s induction (Fig 5, “*N*-glycosylation”). In fact, several *N*-glycosylation sequons displayed positive net site diffsel across all 3 enhanced ER proteostasis environments (Figs 6E, S13E, and S13J). Among those *N*-glycosylation sequons displaying positive net site diffsel, all except N160 are highly variable [68]. These observations add to the evidence that mutational tolerance is more strongly constrained in conserved regions than in variable regions upon upregulation of the host’s ER proteostasis machinery.

The patterns observed for the co-induction of XBP1s and ATF6 largely overlapped with those of XBP1s induction only (S10 and S13A–S13E Figs), except that, with co-induction, CD4 contact sites exhibited a statistically significant decrease in mutational tolerance whereas subunit contact sites did not. Consistent with the less striking reduction in mutational tolerance observed upon ATF6 induction (Fig 2C), we observed that the impact of ATF6 induction was minimal across Env sites when we assessed structural/functional groups independently (S11 and S13F–S13J Figs). Only the gp41 subunit exhibited a small, yet statistically significant, decrease in mutational tolerance (S11 Fig, “Subunits”), which agrees with our slope analysis of the sequence logo plots (Fig 4B–4D).

**Fig 7:**
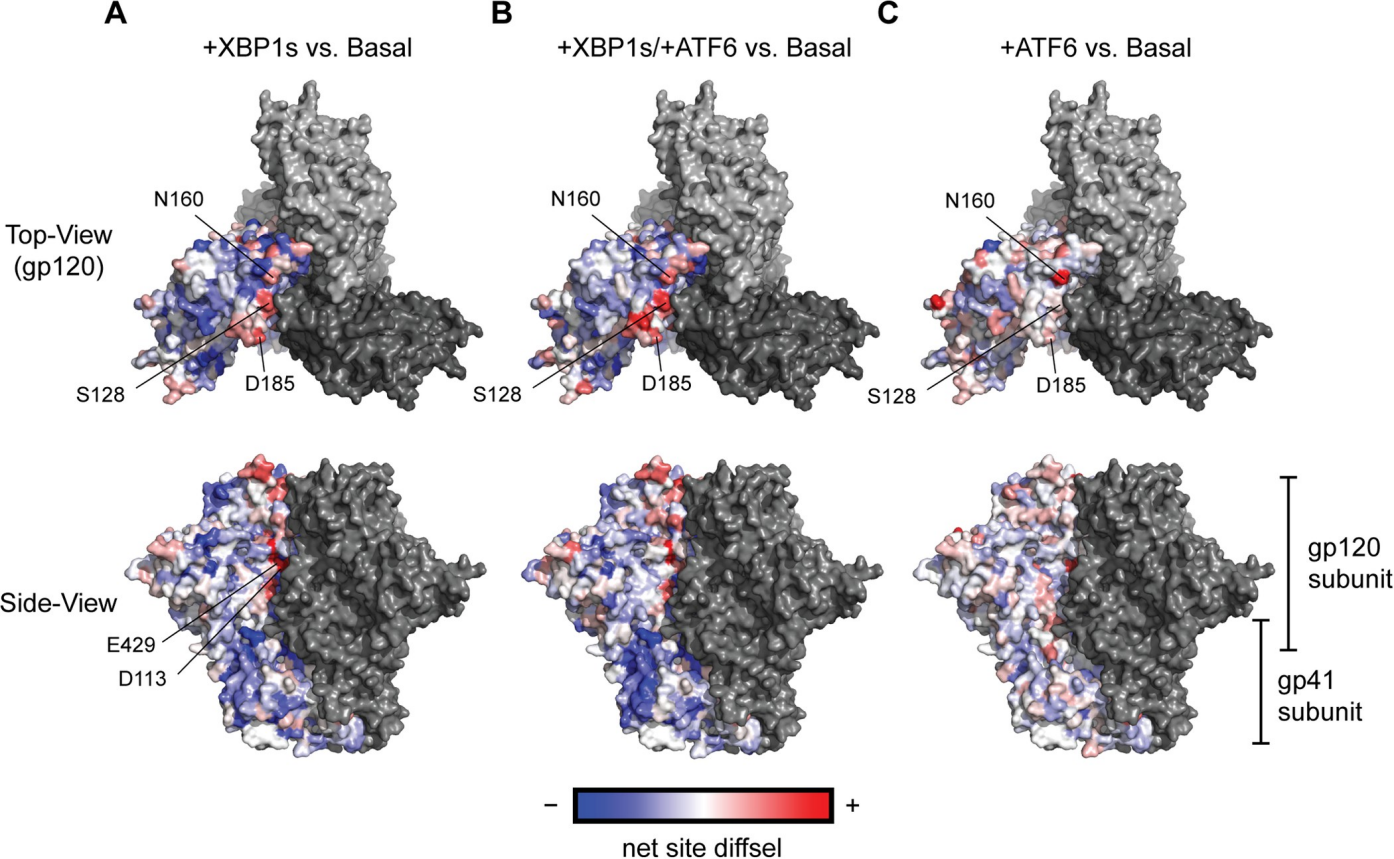
Env sites with positive net site differential selection (diffsel) are clustered at the trimer apex. Average net site diffsel values across Env for (A) +XBP1s (B) +XBP1s/+ATF6, and (C) +ATF6, normalized to the basal endoplasmic reticulum proteostasis environment, are mapped onto Env trimer crystal structure (PDB ID: 5FYK) [73]. One monomer is colored using net site diffsel as the color spectrum; negative net site diffsel residues are colored in blue, and positive net site diffsel residues are colored in red. The remainder of the Env trimer is colored in grey. Diffsel values are provided at https://github.com/yoon-jimin/2021_HIV_Env_DMS.

Finally, to evaluate structural regions whose mutational tolerance was particularly impacted by host ER proteostasis network remodeling, we mapped net site diffsel values onto the Env crystal structure (Fig 7). Whereas mutationally intolerant sites were distributed throughout the Env trimer, sites with enhanced mutational tolerance upon XBP1s induction were located primarily at the apex of the Env trimer (Fig 7A). For instance, N160, S128, and D185 were among the sites with the highest positive net site diffsel. Indeed, although the magnitude of enhanced mutational tolerance varied, these sites exhibited positive net site diffsel in all host ER proteostasis conditions tested. N160, S128, and D185 had similar net site diffsel values when XBP1s was induced (Fig 7A) or when XBP1s and ATF6 were co-induced (Fig 7B), but N160 exhibited substantially higher mutational tolerance when ATF6 was induced (Fig 7C). Notably, N160 belongs to the V2 apex, a well-characterized epitope targeted by the broadly neutralizing antibodies PG9 [69], CH01 [70], CAP256.09 [71], and PGT145 [72], and elimination of the N160 glycan was shown to confer antibody escape [37]. In addition, I165K, a fusion peptide inhibitor resistance mutation [70], was the single variant with the highest positive diffsel when XBP1s and ATF6 were co-induced and the third highest positive diffsel when XBP1s was induced alone, and was also confirmed in our immunoblots to not disrupt Env processing (Fig 3F). These observations suggest that upregulation of host ER proteostasis factors, although generally constraining Env mutational tolerance, can still strongly enhance mutational tolerance in regions of the Env protein in which adaptive mutations are essential, including mutations at certain antibody- or drug-targeted regions of Env.

## Discussion

Our results provide the first experimental evidence, to our knowledge, that UPR-mediated upregulation of the ER proteostasis network can globally reduce the mutational tolerance of a client protein. The primary ER proteostasis factors involved in driving this effect in Env are XBP1s-regulated, as the broadscale effects of ATF6 induction are more muted (Fig 2C and 2F). This result agrees with our RNA-Seq data, where XBP1s induction led to upregulation of a larger number of ER proteostasis factors, including those known to interact with Env [24–28].

The decrease in the mutational tolerance of Env upon ER proteostasis upregulation is consistent with the impacts of cellular quality control factors on protein mutational tolerance, where the available protein sequence space is restricted through degradation and reduced trafficking of aberrantly folded protein variants [16–18]. Previous studies established that Env is readily targeted to and degraded by ERAD [27,28,74], suggesting that destabilizing Env variants may be subjected to more rapid removal by quality control factors in an enhanced ER proteostasis environment. Indeed, upon induction of XBP1s, which upregulates many quality control and ERAD components, conserved regions of Env exhibit particularly large decreases in mutational tolerance (Figs 5 and S10), where mutations are more likely to cause protein misfolding. Rosetta ΔΔ*G* predictions and immunoblotting experiments (Fig 3) confirmed that the variants with negative diffsel upon XBP1s induction were generally more destabilizing and exhibited larger processing defects than the positive diffsel variants.

While our evidence is consistent with the notion that UPR-regulated quality control factors are moderating Env mutational tolerance directly, some of the observed effects could also be secondary. For example, ER proteostasis factors could post-translationally influence the folding or levels of endogenous proteins that regulate Env function or folding. Both of these phenomena are interesting. In addition, we note that the LAI strain of HIV used in this study could have lower mutational tolerance than HIV strains on average, as it was isolated from a chronically infected individual and potentially accumulated a significant number of deleterious mutations. In future studies, it will be interesting to examine effects in additional HIV strains.

This work augments the emerging evidence that host ER proteostasis machinery can fundamentally define the mutational tolerance of viral membrane proteins. Prior to this study, the consequences of ER proteostasis network composition for the mutational tolerance of a membrane protein, whether viral or endogenous, had only ever been investigated for one other protein—influenza hemagglutinin [32]. We show that the host ER proteostasis network also impacts Env mutational tolerance, implying the possibility that this relationship is applicable across multiple RNA viruses and diverse membrane proteins. Moreover, the present work reveals that the interaction between host proteostasis and viral proteins is highly nuanced, and the outcome can differ for each viral pathogen, either because of intrinsic differences in the client protein or because of differences in the cell types the viruses infect. For example, hemagglutinin mutational tolerance is enhanced at febrile temperatures (39°C) upon XBP1s induction, with very minimal effects at a permissive temperature (37°C) [32]. Unlike hemagglutinin, the majority of Env sites exhibited strongly decreased mutational tolerance upon upregulation of host ER proteostasis factors, in this case even at a permissive temperature. Comparing our RNA-Seq data from SupT1^DAX^ cells with a previous characterization of the HEK293^DAX^ cells used in the hemagglutinin work [32], we observe that 73% (+XBP1s) and 58% (+XBP1s/+ATF6) of the transcripts upregulated in SupT1^DAX^ cells were also upregulated in the HEK293^DAX^ cells (note that the +ATF6 condition was not tested in the previous study; S11 Data). Differences in the UPR response in the 2 cell lines, as well structure and folding pathway differences in hemagglutinin and Env themselves, may underpin the differing observations. Although beyond the scope of this paper, a comparative analysis of the interactomes of hemagglutinin and Env with UPR-regulated ER proteostasis factors, particularly focusing on the genes that were differentially enriched in HEK293^DAX^ and SupT1^DAX^ cells, may reveal specific contributors to this differing outcome.

Looking deeper into our observations for Env itself, this study highlights several Env regions that merit further investigation with respect to their roles in Env folding and structure. For example, we found that sites that constitute *N*-glycosylation sequons exhibited both positive and negative net site diffsel (Figs 6E, S13E, and S13J). The fact that *N*-glycosylated residues were not particularly constrained may reflect that they can act redundantly in endowing key interactions with lectin-based chaperone and quality control pathways. Indeed, we previously showed that a nonnative *N*-glycosylation sequon can successfully enable calnexin/calreticulin-mediated ER client protein folding [67]. In addition, while *N*-glycans in Env are important for antibody shielding and viral replication [75–78], there have been varying reports on whether the majority of the *N*-glycans are required for proper folding of Env [78,79]. The specific *N*-glycan sites that proved particularly sensitive to XBP1s upregulation are likely to play some important role in the folding, quality control, and/or trafficking of Env. It will be interesting to explore the specific biophysical mechanisms underlying our observations in future work.

Finally, we find that different sites within a single viral protein can respond differently to the selection pressure imposed by the host ER proteostasis network (Figs 5, 6, S7, S8, and S13). Contrary to the global tendency towards decreased mutational tolerance, we observed many Env sites with positive net site diffsel, especially at the trimer apex of Env (Fig 7). We discovered that N160—where a glycan is installed that is obligatory for binding of the vast majority of V2 apex broadly neutralizing antibodies [80]—exhibited enhanced mutational tolerance in all 3 proteostasis environments and particularly when ATF6 was induced alone. We also observed that I165K, an Env variant known to be fusion peptide inhibitor resistant [70], exhibited highly positive diffsel upon XBP1s induction. These observations indicate that, although the majority of Env sites exhibited depletion of variants, important antibody- or drug-escape variants may be enriched upon upregulation of host ER proteostasis network mechanisms. Thus, the host ER proteostasis environment can strongly influence the mutational tolerance of specific Env variants that are of therapeutic interest.

In conclusion, our results establish that stress-response-mediated upregulation of proteostasis networks can actually restrict rather than increase accessible client protein sequence space, in contrast to most prior work focused on the effects of individual chaperones. We also find that evolutionary interactions between viral proteins and host proteostasis factors are specific to the virus type, as well as to specific regions of the viral protein. We anticipate that this knowledge will prove particularly valuable for ongoing efforts to target host proteostasis network components for antiviral therapeutics [52,81–86] and for the design of proteostasis-network-targeted therapeutic adjuvants that can prevent the emergence of viral variants that confer immune system escape or drug resistance. More broadly, the principles observed here seem likely to prove generally applicable not just to viral proteins but also to endogenous client proteins.

## Materials and methods

### Cell culture

Human T lymphoblasts (SupT1 cells; ATCC) were grown in RPMI-1640 medium (Corning), supplemented with 10% heat-inactivated fetal bovine serum (FBS, Cellgro) and 1% penicillin/streptomycin/glutamine (Cellgro) at 37°C with 5% CO_2_ (g). TZM-bl reporter cells (NIH AIDS Research and Reference Reagent Program; Cat. no. 1470) and HEK293T cells were cultured in DMEM (Corning) supplemented with 10% FBS and 1% penicillin/streptomycin/glutamine at 37°C with 5% CO_2_ (g). Cell lines were periodically tested for mycoplasma using the MycoSensor PCR Assay Kit (Agilent).

### Transfection of HEK293T cells with Env

For transient expression of Env in HEK293T cells, we used the Env gene from HIV-LAI in a pcDNA3.1 expression vector (Addgene) [87]. Env variants were introduced by site-directed mutagenesis (Agilent) and confirmed by Sanger sequencing of the Env gene (S1 Table). HEK293T cells were plated in 6-well plates at a density of 7 × 10^5^ cells/well and allowed to adhere overnight. The next day, cells were transfected with 1.5 μg of eGFP in pcDNA3.1 (GFP control) or 0.15 μg of eGFP and 1.35 μg of Env plasmid using Lipofectamine reagents (Thermo Fisher). After 16 h, the medium was changed. After another 24 h, cells were harvested for analysis.

### Plasmids to engineer SupT1^DAX^ cells

The following lentiviral destination vectors were used for stable cell line construction: pLenti6/V5-DEST Gateway with a tetracycline repressor insert (Invitrogen) and blasticidin resistance, pLenti CMV/TO Zeocin DEST with either human XBP1s insert (Addgene), and pLenti CMV Hygromycin DEST with a DHFR.ATF6(1–373) fusion, as previously described [41].

### Stable cell line engineering

We generated a stable SupT1^DAX^ cell line using a previously described method for chemical genetic control of IRE1-XBP1s and ATF6 transcription factors [41]. Specifically, SupT1 cells were first transduced with lentivirus encoding a blasticidin-resistant tetracycline repressor and then with lentivirus encoding zeocin-resistant XBP1s. Transduction was performed by spinoculation with 2 μg/mL polybrene (Sigma-Aldrich) at 1,240*g* for 1–1.5 h. Heterostable cell lines expressing the tetracycline repressor and XBP1s were then selected using 10 μg/mL blasticidin (Gibco) and 50 μg/mg zeocin (Invitrogen). Single colony lines were derived from the heterostable population by seeding 30–40 cells in a 96-well plate in 100 μl of RPMI medium without antibiotics for 10–14 d. Clonal populations were then selected and expanded in 24-well plates in 500 μL of RPMI containing 10 μg/mL blasticidin and 50 μg/mL zeocin. Cells were grown to confluency and then screened based on functional testing of the XBP1s construct using real-time reverse transcription polymerase chain reaction (RT-PCR; described below) with or without 2 μg/mL dox (Alfa Aesar). The selected SupT1 single colony cell line encoding tetracycline-inducible XBP1s was then transduced with lentivirus encoding DHFR.ATF6(1–373) via the spinoculation protocol described above, and stable cells were selected using 400 μg/mL hygromycin B (Gibco). The heterostable populations were then treated with vehicle, 2 μg/mL dox, 10 μM TMP (Alfa Aesar), or 2 μg/mL dox and 10 μM TMP and screened for function using RT-PCR (described below) to obtain the final stably engineered SupT1^DAX^ cell line.

### RT-PCR

For RT-PCR of SupT1 cells to screen for stably engineered SupT1^DAX^ cells with desired properties, SupT1^DAX^ cells were seeded at a density of 2 × 10^5^ cells/well in a 6-well plate in RPMI medium and treated with 0.01% DMSO, 2 μg/mL dox, 10 μM TMP, or 2 μg/mL dox and 10 μM TMP for 18 h. As a positive control for UPR induction, the cells were treated with 10 μg/mL tunicamycin (Sigma-Aldrich) for 6 h. Cellular RNA was harvested using the Omega RNA Extraction Kit with Homogenizer Columns (Omega Bio-tek). 1 μg of RNA was used to prepare cDNA using random primers (total reaction volume = 20 μL; Applied Biosystems High-Capacity Reverse Transcription Kit). The reverse transcription reaction was diluted to 80 μL with water, and 2 μL of each sample was used for qPCR with 2× SYBR Green (Roche) and primers for human *RPLP2* (housekeeping gene), *HSPA5* (BiP), *HSP90B1* (GRP94), *DNAJB9* (ERDJ4), and *SEC24D* (S1 Table). For qPCR data analysis, all gene transcripts were normalized to that of *RPLP2*, and the fold change in expression relative to DMSO-treated cells was calculated.

For RT-PCR of HEK293T cells, cells were transfected with Env variants, and cellular RNA was harvested using the Omega RNA Extraction Kit with Homogenizer Columns. As a positive control for UPR induction, GFP-transfected cells were treated with 2 μM thapsigargin (Sigma-Aldrich) for 6 h prior to RNA harvest. The reverse transcription reaction was performed identically as in SupT1 cells, and 2 μL of each sample was used for qPCR with 2× SYBR Green and primers for human *RPLP2* (housekeeping gene), *SEC24D*, *HSPA5* (BiP), *DNAJB9* (ERDJ4), and *HYOU1* (S1 Table). For qPCR data analysis, all gene transcripts were normalized to that of *RPLP2*, and the fold change in expression relative to GFP-transfected cells was calculated.

### RNA-Seq

SupT1^DAX^ cells were seeded in a 6-well plate at a density of 5 × 10^5^ cells/well in RPMI medium in quadruplicate. The cells were treated with 0.01% DMSO (vehicle), 2 μg/mL dox (to activate the XBP1s transcriptional response), 10 μM TMP (to activate the ATF6 transcriptional response), or 2 μg/mL dox and 10 μM TMP (to simultaneously activate the XBP1s and ATF6 transcriptional responses) for 24 h. Cellular RNA was harvested using the RNeasy Plus Mini Kit with QIAshredder homogenization columns (Qiagen). RNA-Seq libraries were prepared using the Kapa mRNA HyperPrep RNA-Seq library construction kit (Kapa/Roche), with 6 min of fragmentation at 94°C and 9 PCR cycles of final amplification and duplex barcoding. Libraries were quantified using the Fragment Analyzer and qPCR before being sequenced on an Illumina HiSeq 2000 using 40-bp single-end reads in high output mode.

Analyses were performed using previously described tools and methods [88]. Reads were aligned against hg19 (February 2009) using BWA mem v. 0.7.12-r1039 (RRID:SCR_010910) with flags –t 16 –f, and mapping rates, fraction of multiply-mapping reads, number of unique 20-mers at the 5′ end of the reads, insert size distributions, and fraction of ribosomal RNAs were calculated using BEDTools v. 2.25.0 (RRID:SCR_006646) [89]. In addition, each resulting bam file was randomly down-sampled to a million reads, which were aligned against hg19, and read density across genomic features was estimated for RNA-Seq-specific quality control metrics. For mapping and quantitation, reads were aligned against GRCh38/ENSEMBL 89 annotation using STAR v. 2.5.3a with the following flags -runThreadN 8 –runMode alignReads –outFilterTyp BySJout –outFilterMultimapNmax 20 –alignSJoverhangMin 8 –alignSJDBoverhangMin 1 –outFilterMismatchNmax 999 –alignIntronMin 10 –alignIntronMax 1000000 –alignMatesGapMax 1000000 –outSAMtype BAM SortedByCoordinate –quantMode TranscriptomeSAM pointing to a 75-nt junction GRCh38 STAR suffix array [90]. Gene expression was quantitated using RSEM v. 1.3.0 (RRID:SCR_013027) with the following flags for all libraries: rsem-calculate-expression–calc-pme–alignments–p 8 –forward-prob 0 against an annotation matching the STAR SA reference [91]. Posterior mean estimates (PMEs) of counts and estimated RPKM were retrieved.

For differential expression analysis, dox-, TMP-, or dox- and TMP-treated SupT1^DAX^ cells were compared against vehicle-treated SupT1^DAX^ cells. Differential expression was analyzed in the R statistical environment (R v.3.4.0) using Bioconductor’s DESeq2 package on the protein-coding genes only (RRID:SCR_000154) [92]. Dataset parameters were estimated using the estimateSizeFactors and estimateDispersions functions; read counts across conditions were modeled based on a negative binomial distribution, and a Wald test was used to test for differential expression (nbinomWaldtest, all packaged into the DESeq function), using the treatment type as a contrast. Shrunken log_2_ fold changes were calculated using the lfcShrink function. Fold changes and *p*-values are reported for each protein-coding gene. Upregulation was defined as a change in expression level > 1.5-fold relative to the basal environment with a non-adjusted *p*-value < 10^−10^. Gene ontology analyses were performed using the online DAVID server, according to tools and methods presented by Huang et al. [88]. The volcano plots were generated using EnhancedVolcano (Fig 1B–1D; https://github.com/kevinblighe/EnhancedVolcano).

### Gene set enrichment analysis (GSEA)

Differential expression results from DESeq2 were retrieved, and the “stat” column was used to pre-rank genes for GSEA. These “stat” values reflect the Wald test performed on read counts as modeled by DESeq2 using the negative binomial distribution. Genes that were not expressed were excluded from the analysis. GSEA (desktop version, v3.0) [47,93] was run in the pre-ranked mode against the MSigDB 7.0 C5 (Gene Ontology) set, using the official gene symbol as the key, with a weighted scoring scheme, normalizing by meandiv, with 8,958 gene sets retained, and 5,000 permutations were run for *p*-value estimation. Selected enrichment plots were visualized using a modified version of ReplotGSEA, in R (https://github.com/PeeperLab/Rtoolbox/blob/master/R/ReplotGSEA.R).

### Resazurin metabolism assay

SupT1^DAX^ cells were seeded in 96-well plates (Corning) at a density of 1.5 × 10^5^ cells/well in RPMI medium and then treated with 0.1% DMSO, 2 μg/mL dox, 10 μM TMP, or 2 μg/mL dox and 10 μM TMP. 72 h post-treatment, 50 μL of RPMI containing 0.025 mg/mL resazurin sodium salt (Sigma) was added to the wells and mixed thoroughly. After 2 h of incubation, resorufin fluorescence (excitation 530 nm; emission 590 nm) was quantified using a Take-3 plate reader (BioTeK). Experiments were conducted in biological quadruplicate.

### HIV titering

TZM-bl reporter cells were seeded at a density of 2.5 × 10^4^ cells/well in 48-well plates. After 5 h, the cells were infected with 100 μL of serially diluted infectious HIV viral inoculum containing 10 μg/ml polybrene. Each sample was used to infect 4 technical replicates. After 48 h, the viral supernatant was removed, and the cells were washed twice with PBS and then fixed with 4% paraformaldehyde (Thermo Scientific) for 20 min. The fixed cells were washed twice with PBS and then stained with 4 mM potassium ferrocyanide, 4 mM ferricyanide, and 0.4 mg/mL 5-bromo-4-chloro-3-indolyl β-D-galactopyranoside (X-Gal) in PBS at 37°C for 50 min. The cells were washed with PBS, blue cells were counted manually under a microscope, and infectious titers were calculated based on the number of blue cells per volume of viral inoculum.

### Deep mutational scanning

Three biological replicate HIV libraries were generated from 3 previously prepared independent Env mutant plasmid libraries (a generous gift from Prof. Jesse Bloom, University of Washington) following the previously reported protocol [22]. Briefly, to generate the plasmid libraries, codon mutant libraries of *env* were first created via PCR mutagenesis using codon tiling mutagenic primers [55] For each codon except the starting methionine, the N-terminal signal peptide, and the C-terminal cytoplasmic tail, primers with a randomized NNN nucleotide triplet in the codon of interest were used to create the forward- and reverse-mutagenesis primer pool, the 2 fragment PCR reactions were run, and the products were joined with additional PCR reactions. The resulting *env* amplicons were cloned into a recipient plasmid that had *env* replaced by GFP, and transformed into competent cells to prepare the plasmid library. For DMS, SupT1^DAX^ cells were seeded in T175 vented tissue culture flasks (Corning) at a density of 1.0 × 10^8^ cells/flask in RPMI medium. The cells were pre-treated with 0.01% DMSO, 2 μg/mL dox, 10 μM TMP, or 2 μg/mL dox and 10 μM TMP for 18 h. Pre-treated cells were infected with the p1 viral libraries at a MOI of 0.005 based on the infectious (TZM-bl) titers. In addition, 1 flask was either mock-infected (negative control) or infected with wild-type virus (to enable error correction for DMS data analysis). To remove unbound virions from culture, 6 h post infection the cells were pelleted at 2,000*g* for 5 min, washed twice with 25 mL of PBS, and then resuspended in 50 mL of RPMI medium treated with 0.01% DMSO, 2 μg/mL dox, 10 μM TMP, or 2 μg/mL dox and 10 μM TMP. Cell pellets were harvested 96 h post infection by centrifuging the culture at 2,000*g* for 5 min. Cell pellets were washed twice with PBS and then resuspended in 1 mL of PBS. Aliquots (100 μL) were added to Eppendorf tubes and stored at −80°C for subsequent DNA extraction.

To generate samples for Illumina sequencing, non-integrated viral DNA was purified from aliquots of frozen SupT1^DAX^ cells using a mini-prep kit (Qiagen) and approximately 10^7^ cells per prep. PCR amplicons of Env were prepared from plasmid or mini-prepped non-integrated viral DNA by PCR following a previously described protocol [22]. The amplicons were sequenced using barcoded-subamplicon sequencing, dividing Env into 9 rather than the previously reported 6 subamplicons. We note that it was necessary to exclude Env amino acid residues 31–34 from analysis because, after PCR optimization, we were unable to identify functional primers for the first subamplicon that did not include these sites. As previously described, at least 10^6^ Env molecules were PCR-amplified for preparation of subamplicon sequencing libraries to ensure sufficient sampling of viral library diversity [56]. Briefly, this sequencing library preparation method appends unique, random barcodes and part of the Illumina adapter to Env subamplicon molecules. In a second round of PCR, the complexity of the uniquely barcoded subamplicons was controlled to be less than the sequencing depth, and the remainder of the Illumina adapter was appended. The resulting libraries were sequenced on an Illumina HiSeq 2500 in rapid run mode with 2 × 250-bp paired-end reads. The primers used are described in S1 Table.

### DMS data analysis

The software dms_tools2 (https://jbloomlab.github.io/dms_tools2/) [57] was used to align the deep-sequencing reads, count the number of times each codon mutation was observed both before and after selection, calculate the diffsel for each Env variant, and generate sequence logo plots (Figs 4A, S7, and S8). The IPython notebook for code to perform this analysis is provided at https://github.com/yoon-jimin/2021_HIV_Env_DMS. In sequence logo plots, regions with decreased mutational tolerance were defined as regions of Env where there were more than 15 amino acid residues in a row with slope < −1.5 (for +XBP1s and +XBP1s/+ATF6) or slope < −1 (+ATF6) (Fig 4B–4D). The slope at residue *i* was calculated using the following formula:

$$\frac{{\left(cumulative\;net\;site\;diffsel\right)}_{i+5}-{\left(cumulative\;net\;site\;diffsel\right)}_{i-5}}{10}$$

Surface accessible area (SAA) was calculated via PDBePISA (S9 Fig) [94] using the crystal structure of BG505 SOSIP.664 (PDB ID: 5V8M) [95] and aligning to the LAI Env sequence. PDBePISA calculates the solvent-accessible surface area of the monomer (ASA value) and the solvent-accessible surface area that is buried upon formation of interfaces (“buried surface in interfaces” value). “Buried surface in interfaces” values were subtracted from ASA values to obtain the SAA of the trimer. Ligands and antibodies were removed from the PDB file prior to SAA analysis. Site entropy (Shannon entropy) was calculated using the Los Alamos HIV Sequence Database Shannon Entropy-One tool (S12 Fig). The calculation was based on the consensus sequence generated from the 7,590 HIV-1 Env sequences in the Los Alamos HIV Sequence Database (1 sequence per patient up to 2019). The net site diffsel values were mapped onto Env crystal structure (PDB ID: 5FYK) [73] using PyMOL (Fig 7).

### Calculating changes in protein folding free energy upon mutation using Rosetta

The cartesian_ddg application in Rosetta version 3.13 was used to calculate ΔΔ*G* of protein stability upon substitution [58]. To prepare the initial structure for the ΔΔ*G* calculations, a homology model of the HIV-1 envelope protein for the LAI strain was constructed using the Rosetta comparative modeling protocol, RosettaCM [96]. Residues 31–664 of the HIV Env protein from the HIV-1 JR-FL strain (PDB ID: 5FYK, chain G and chain B) were used as the template structure [73,97]. The structure had a truncation at the membrane proximal external region of gp41, and the homology model was constructed for the domains whose coordinate data were available. A Rosetta symmetry definition file was created using the make_symmdef_file application to prepare the HIV Env trimer structure [98]. There were 34 residues whose coordinates were missing in chains G and B of PDB 5FYK, and in the hybridization process, the missing residues in the threaded structure were patched using target sequence-based fragments and ab initio folding [96]. A total of 1,000 models were generated, and the lowest-energy HIV Env trimer model that preserved the 10 disulfide bonds observed in the crystal structure was selected for ΔΔ*G* calculations.

The HIV Env trimer structure was relaxed using the Rosetta FastRelax application, which performed 5 cycles of side-chain repacking and energy minimization using the Rosetta energy function ref2015_cart [58,99–101]. A total of 20 relaxed decoys were generated, and the lowest energy structure was used as the input wild-type structure for the cartesian_ddg calculation. In the cartesian_ddg calculation, the target residue was substituted in all 3 chains of the trimer structure, and any neighboring residues within a 9-Å radius were repacked and energy-minimized using the ref2015_cart energy function. This process was repeated 5 times to produce 5 energy scores for the mutant and for the wild type. The ΔΔ*G* values were calculated by subtracting the average wild-type scores from the average mutant scores. To better relate the predicted ΔΔ*G* values to experimental values, the ΔΔ*G* values were then scaled by a factor of 0.34, which was previously determined by fitting ΔΔ*G* values calculated using Rosetta to experimental ΔΔ*G* values in units of kcal/mole [58]. The resulting ΔΔ*G* values were divided by 3 to obtain the predicted ΔΔ*G* values for 1 monomer of the trimer.

### Immunoblots

For immunoblotting of SupT1^DAX^ cells for UPR target proteins, SupT1^DAX^ cells were seeded in T75 culture flasks in RPMI medium and grown until cells attained a density of 1 × 10^6^ cells/mL. Cells were then treated with 0.01% DMSO (vehicle control), 2 μg/mL dox (+XBP1s), 10 μM TMP (+ATF6), or 2 μg/mL dox and 10 μM TMP (+XBP1s/+ATF6) for 24 h. After treatment, cells were pelleted by centrifugation at 1,000 × g for 5 min. Pellets were washed with 1× PBS, and then lysed in radioimmunoprecipitation assay (RIPA) buffer (25 mM Tris [pH 8.0], 0.5% [m/v] sodium deoxycholate, 150 mM NaCl, 0.1% [m/v] sodium dodecyl sulfate, 1% [v/v] IGEPAL CA-630) and protease inhibitor tablet (Thermo Fisher). Lysates were cleared by centrifugation at 20,000*g* for 20 min, and total protein concentration was quantified using bicinchoninic acid assay (Thermo Fisher); 108 μg of total protein was analyzed for each sample. Blots were incubated with anti-BiP primary (Cell Signaling Technology), anti-SEC24D primary (Abcam), anti-β-actin primary (Sigma), and 680 RD and 800 CW secondary (LI-COR) antibodies, and imaged by scanning on an Odyssey infrared imager (LI-COR).

For immunoblotting of HEK293T cells, cells were transfected with Env variants, pelleted, washed with 1× PBS, and then lysed in RIPA buffer and protease inhibitor tablet. Lysates were cleared by centrifugation at 20,000*g* for 20 min, and total protein concentration was quantified using the bicinchoninic acid assay; 30 μg of total protein was analyzed for each sample. Blots were incubated with anti-gp41 primary (ARP-13049; obtained through the NIH HIV Reagent Program, contributed by Dr. George Lewis) and 680 RD secondary antibodies, and imaged by scanning on an Odyssey infrared imager, followed by quantification using Image Studio.

### Statistical analyses

Unless indicated otherwise, experiments were performed in biological triplicate with replicates defined as independent experimental entireties (i.e., from plating the cells to acquiring the data). For DMS, each biological replicate mutant viral library was prepared from independently generated mutant plasmid libraries, as previously reported [56]. The mean of ΔΔ*G* distributions (Fig 3A and 3B) were tested for significance using a 2-sample *t* test in GraphPad Prism. Densitometric analyses of immunoblots (Fig 3E and 3F) were tested for statistical significance using 1-way ANOVA followed by Dunnett’s test in GraphPad Prism, comparing the mean of each variant to the mean of wild type. Diffsel values from DMS were tested for significance of deviation from 0 (no relative enrichment or depletion), using a 1-sample *t* test in GraphPad Prism. For diffsel values and net site diffsel values, 2-tailed *p*-values are reported to assess whether the mean (net site) diffsel values for enhanced ER proteostasis environments were significantly different from 0 (Fig 2C and 2F). For net site diffsel distributions for specific functional and structural groups, *p*-values were Bonferroni-corrected for 20 tests (Figs 5, S10, and S11).

## Supporting information

S1 Data Complete RNA-Seq differential expression analysis. (XLSX)

S2 Data Complete GSEA. (XLSX)

S3 Data Resazurin assay and TZM-bl assay. (XLSX)

S4 Data. Library coverage (XLSX)

S5 Data Complete ΔΔ*G* analysis data. (XLSX)

S6 Data Immunoblot densitometric analysis. (XLSX)

S7 Data RT-PCR of UPR genes upon transfection of Env variants. (XLSX)

S8 Data Cumulative net site diffsel. (XLSX)

S9 Data Surface accessible area. (XLSX)

S10 Data Site entropy. (XLSX)

S11 Data Transcriptome comparison of HEK293^DAX^ cells and SupT1^DAX^ cells. (XLSX)

S1 Fig Immunoblot of SupT1^DAX^ cells shows that the XBP1s and ATF6 pathways are successfully and differentially induced. Representative immunoblot image showing specific upregulation of XBP1s (Sec24D) and ATF6 (BiP) protein targets in SupT1^DAX^ cells upon vehicle treatment (basal), dox treatment (+XBP1s), TMP treatment (+ATF6), and co-treatment of dox and TMP (+XBP1s/+ATF6).
(TIF)

S2 Fig ER proteostasis perturbation has no deleterious effects on cell viability and does not restrict HIV replication. (A) Induction of XBP1s, induction of ATF6, or co-induction of XBP1s and ATF6 did not alter the metabolic activity of SupT1 cells, as measured by a resazurin assay. The average of biological quadruplicates is plotted, with error bars representing the standard deviation. Individual data points are also shown. (B) Induction of XBP1s and co-induction of XBP1s and ATF6 did not restrict, and actually slightly increased, HIV infectious titers, while induction of ATF6 did not influence HIV replication in SupT1 cells, as measured by TZM-bl infectious units. The average of biological triplicates is plotted, with error bars representing the standard deviation. Individual data points are also shown. For (A) and (B), replicate data are provided in S3 Data.
(TIF)

S3 Fig Library coverage was generally consistent throughout the Env sequence. The number of codons observed fewer than 3 times after summing the codon counts over the 3 biological replicate libraries is plotted against the amino acid site number. Sites with lower coverage were not localized to any specific domain of structural or functional importance. Data values for library coverage are provided in S4 Data.
(TIF)

S4 Fig Subamplicon sequencing strategy ensures greater accuracy of reads during deep sequencing. The full-length Env gene was divided into 9 subamplicons. In the first round of PCR, unique, random barcodes and part of the Illumina adapter were appended to the Env subamplicon molecules. In the second round of PCR, the complexity of the uniquely barcoded subamplicons was controlled to be less than the sequencing depth, and the remainder of the Illumina adapter was appended. The resulting libraries were sequenced on an Illumina HiSeq 2500 in rapid run mode with 2 × 250-bp paired-end reads.
(TIF)

S5 Fig Env variants with negative diffsel exhibit processing defects. Immunoblots in biological triplicates showing gp160 and gp41 bands for selected variants with (A) negative diffsel and (B) positive diffsel upon XBP1s induction.
(TIF)

S6 Fig Transient transfection of Env variants with highly negative diffsel does not induce UPR. RT-PCR analysis of *SEC24D*, *HSPA5*, *DNAJB9*, and *HYOU1* in HEK293T cells expressing GFP (negative control), wild-type Env, and 3 Env variants that were strongly negatively selected in +XBP1s versus basal (C54W, L111P, and L556R). As a positive control for UPR induction, HEK293T cells expressing GFP were treated with thapsigargin (Tg; 2 μM) for 6 h (GFP + Tg). RT-PCR data are presented as fold increase relative to GFP-transfected negative control. RT-PCR data values are provided in S7 Data.
(TIF)

S7 Fig Sequence logo plots reveal diffsel across Env upon co-induction of XBP1s and ATF6. Logo plot displaying averaged diffsel for +XBP1s/+ATF6 normalized to the basal proteostasis environment. The height of the amino acid abbreviation corresponds to the magnitude of diffsel. The amino acid abbreviations are colored based on their side-chain properties: negatively charged (D, E; red), positively charged (H, K R; blue), polar uncharged (C, S, T; orange/N, Q; purple), small nonpolar (A, G; pink), aliphatic (I, L, M, P, V; green), and aromatic (F, W, Y; brown). The numbers and letters below the logos indicate the Env site in HXB2 numbering and the identity of the wild-type amino acid for that site, respectively. The color bar below the logos indicates the function (F) that the site is involved in (*N*-glycosylation site [purple], disulfide bond [green], or salt bridge [red]) or the region (R) of Env that the site belongs to (gp120–variable [purple], gp120–conserved [cyan], gp41 [yellow], or transmembrane domain [red]; the sites that belong to the 5 variable loops of gp120 were categorized as “gp120–variable,” and the sites that are not included in the 5 variable loops were categorized as “gp120–conserved”). Only variants that were present in all 3 pre-selection viral libraries and exhibited diffsel in the same direction across all 3 biological triplicates are plotted here. Diffsel values as well as unfiltered logo plots for each individual replicate are provided at https://github.com/yoon-jimin/2021_HIV_Env_DMS.
(TIF)

S8 Fig Sequence logo plots reveal diffsel across Env upon induction of ATF6. Logo plot displaying averaged diffsel for +ATF6 normalized to the basal proteostasis environment. The height of the amino acid abbreviation corresponds to the magnitude of diffsel. The amino acid abbreviations are colored based on their side-chain properties: negatively charged (D, E; red), positively charged (H, K R; blue), polar uncharged (C, S, T; orange/N, Q; purple), small nonpolar (A, G; pink), aliphatic (I, L, M, P, V; green), and aromatic (F, W, Y; brown). The numbers and letters below the logos indicate the Env site in HXB2 numbering and the identity of the wild-type amino acid for that site, respectively. The color bar below the logos indicates the function (F) that the site is involved in (*N*-glycosylation site [purple], disulfide bond [green], or salt bridge [red]) or the region (R) of Env that the site belongs to (gp120–variable [purple], gp120–conserved [cyan], gp41 [yellow], or transmembrane domain [red]; the sites that belong to the 5 variable loops of gp120 were categorized as “gp120–variable,” and the sites that are not included in the 5 variable loops were categorized as “gp120–conserved”). Only variants that were present in all 3 pre-selection viral libraries and exhibited diffsel in the same direction across all 3 biological triplicates are plotted here. Diffsel values as well as unfiltered logo plots for each individual replicate are provided at https://github.com/yoon-jimin/2021_HIV_Env_DMS.
(TIF)

S9 Fig Env net site diffsel is not correlated with surface accessible area (SAA). Average net site diffsel values plotted against the SAA of Env monomer (A–C) and trimer (D–F). Average net site diffsel values for +XBP1s (A and D), +ATF6 (B and E), and +XBP1s/+ATF6 (C and F) were normalized to the basal ER proteostasis environment and plotted against the SAA at each site. The percentages of variants with positive and negative net site diffsel for the left and right half of the plot are stated, as well as the Pearson correlation coefficient *r*. SAA was calculated using PDBePISA [94] with PDB ID 5V8M [95], where SAA = 0 corresponds to a buried site. SAA data values are provided in S9 Data.
(TIF)

S10 Fig Impact of combined induction of XBP1s and ATF6 on mutational tolerance varies across Env structural elements. Average net site diffsel for the +XBP1s/+ATF6 ER proteostasis environment normalized to the basal ER proteostasis environment, where the means of distributions are indicated by black horizontal lines. Sites are sorted by TMD versus soluble, subunits, conserved versus variable regions of gp120, the 5 variable loops of gp120, regions important for membrane fusion, and other structural/functional groups. For TMD versus soluble, all sites that do not belong to the TMD were categorized as “soluble.” For conserved versus variable, the sites that belong to the 5 variable loops of gp120 were categorized as “gp120–variable,” and the sites that are not included in the 5 variable loops were categorized as “gp120–conserved.” Significance of deviation from null (net site diffsel = 0, no selection) was tested using a 1-sample *t* test. The derived *p*-values were Bonferroni-corrected for 20 tests;*p-value < 0.05, **p-value < 0.01, ***p-value < 0.001, ****p-value < 0.0001; ns, not significant. Diffsel values are provided at https://github.com/yoon-jimin/2021_HIV_Env_DMS. Assignments for these structural regions are provided in S2 Table.
(TIF)

S11 Fig Impact of ATF6 induction on mutational tolerance varies across Env structural elements. Average net site diffsel for the +ATF6 ER proteostasis environment normalized to the basal ER proteostasis environment, where the means of distributions are indicated by black horizontal lines. Sites are sorted by TMD versus soluble, subunits, conserved versus variable regions of gp120, the 5 variable loops of gp120, regions important for membrane fusion, and other structural/functional groups. For TMD versus soluble, all sites that do not belong to the TMD were categorized as “soluble.” For conserved versus variable, the sites that belong to the 5 variable loops of gp120 were categorized as “gp120–variable,” and the sites that are not included in the 5 variable loops were categorized as “gp120–conserved.” Significance of deviation from null (net site diffsel = 0, no selection) was tested using a 1-sample *t* test. The derived *p*-values were Bonferroni-corrected for 20 tests; ****p-value < 0.0001; ns, not significant. Diffsel values are provided at https://github.com/yoon-jimin/2021_HIV_Env_DMS. Assignments for these structural regions are provided in S2 Table.
(TIF)

S12 Fig Enhanced mutational tolerance is observed more frequently at sites with high site entropy. Average net site diffsel values across Env for (A) +XBP1s (B) +ATF6, and (C) +XBP1s/+ATF6 are normalized to the basal ER proteostasis environment and plotted against the site entropy at each site. The percentages of variants with positive and negative net site diffsel for the left and right half of the plot are stated, as well as the Pearson correlation coefficient *r*. Site entropy data values are provided in S10 Data.
(TIF)

S13 Fig Diverse functional elements of Env respond differently to combined induction of XBP1s and ATF6 and induction of ATF6. Selected sequence logo plots for the +XBP1s/+ATF6 (A–E) and +ATF6 (F–J) ER proteostasis environments normalized to the basal ER proteostasis environment for (A and F) the conserved GPGR motif of the V3 loop, (B and G) the hydrophobic patch of the V3 loop, (C and H) the hydrophobic network of gp120 (important for CD4 binding), (D and I) cysteine residues participating in disulfide bonds, and (E and J) selected *N*-glycosylation sequons (N-X-S/T) that exhibited positive net site diffsel in all 3 remodeled proteostasis environments. The height of the amino acid abbreviation corresponds to the magnitude of diffsel. The numbers and letters below the logos indicate the Env site in HXB2 numbering and the wild-type amino acid for that site, respectively. Only variants that were present in all 3 pre-selection viral libraries and exhibited diffsel in the same direction across the biological triplicates are plotted. All logo plots were generated on the same scale. Diffsel values are provided at https://github.com/yoon-jimin/2021_HIV_Env_DMS. Assignments for these functional regions are provided in S2 Table.
(TIF)

S1 Raw Images (TIF)

S1 Table Primers for Env sequencing, RT-PCR, and site-directed mutagenesis. (XLSX)

S2 Table Complete citations for structural and functional groups. (XLSX)

## Abbreviations

diffsel: differential selection
DMS: deep mutational scanning
dox: doxycycline
ER: endoplasmic reticulum
ERAD: endoplasmic-reticulum-associated degradation
GSEA: gene set enrichment analysis
MOI: multiplicity of infection
RNA-Seq: RNA sequencing
RT-PCR: reverse transcription polymerase chain reaction
SAA: surface accessible area
TMD: transmembrane domain
TMP: trimethoprim
UPR: unfolded protein response
