# Supplementary figures and images for: The endoplasmic reticulum proteostasis network profoundly shapes the protein sequence space accessible to HIV envelope
Source: PLoS Biol. 2022 Feb 18;20(2):e3001569. doi: 10.1371/journal.pbio.3001569 (PMC8906867; doi:10.1371/journal.pbio.3001569)

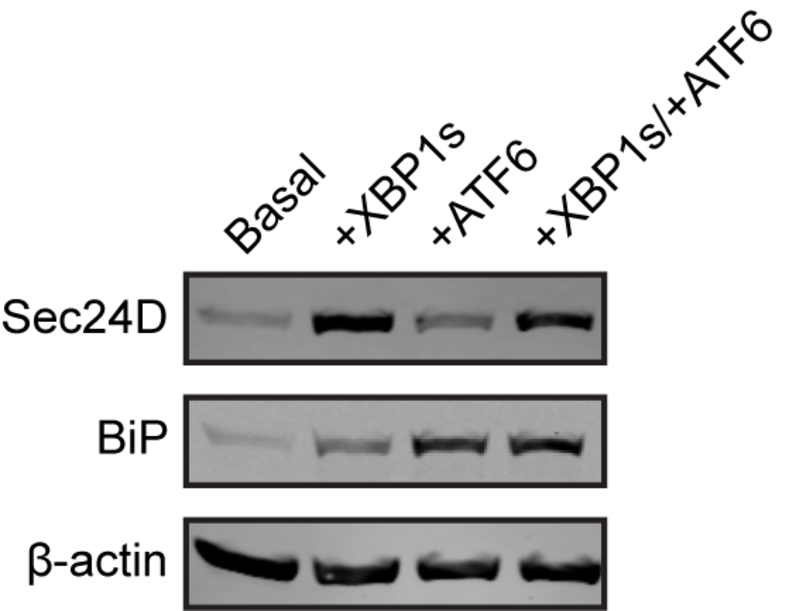

Supplement: S1 Fig — Representative immunoblot image showing specific upregulation of XBP1s (Sec24D) and ATF6 (BiP) protein targets in SupT1DAX cells upon vehicle treatment (basal), dox treatment (+XBP1s), TMP treatment (+ATF6), and co-treatment of dox and TMP (+XBP1s/+ATF6). (TIF) [file pbio.3001569.s012.tif]

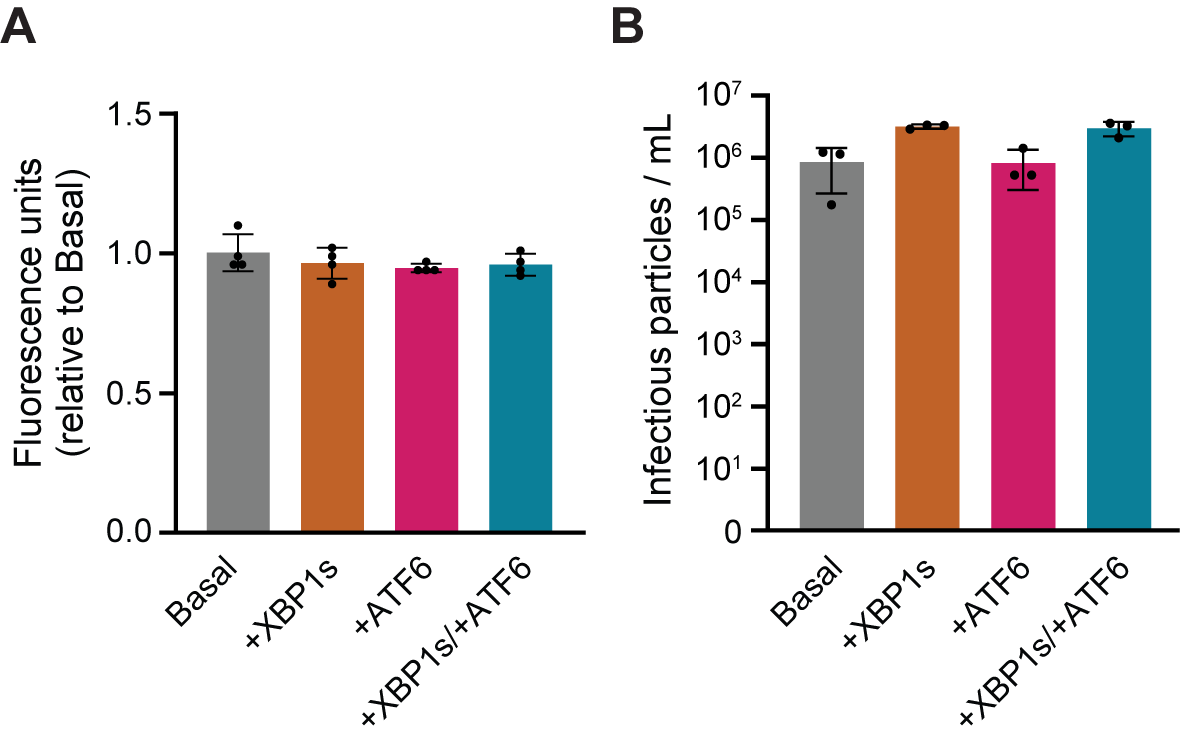

Supplement: S2 Fig — (A) Induction of XBP1s, induction of ATF6, or co-induction of XBP1s and ATF6 did not alter the metabolic activity of SupT1 cells, as measured by a resazurin assay. The average of biological quadruplicates is plotted, with error bars representing the standard deviation. Individual data points are also shown. (B) Induction of XBP1s and co-induction of XBP1s and ATF6 did not restrict, and actually slightly increased, HIV infectious titers, while induction of ATF6 did not influence HIV replication in SupT1 cells, as measured by TZM-bl infectious units. The average of biological triplicates is plotted, with error bars representing the standard deviation. Individual data points are also shown. For (A) and (B), replicate data are provided in S3 Data. (TIF) [file pbio.3001569.s013.tif]

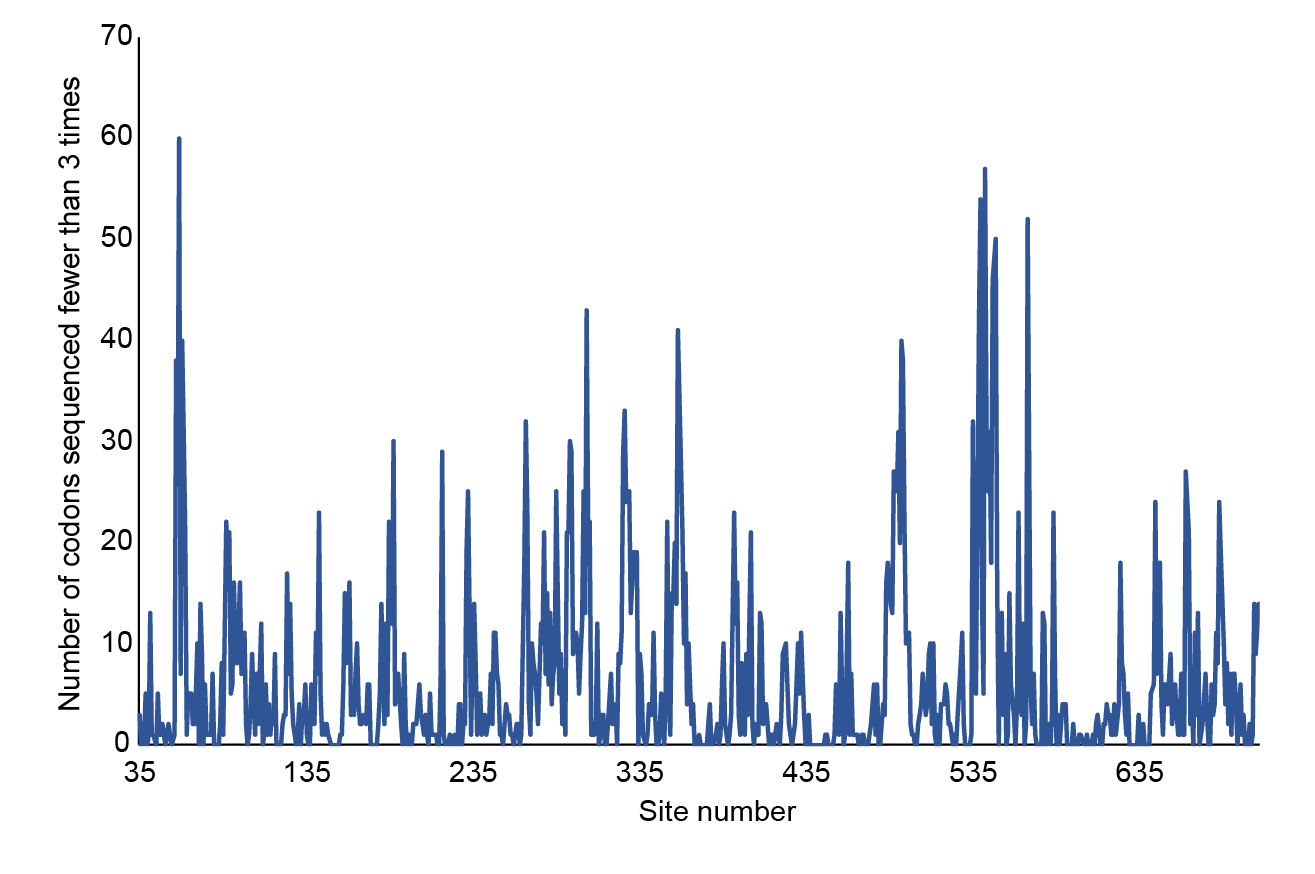

Supplement: S3 Fig — The number of codons observed fewer than 3 times after summing the codon counts over the 3 biological replicate libraries is plotted against the amino acid site number. Sites with lower coverage were not localized to any specific domain of structural or functional importance. Data values for library coverage are provided in S4 Data. (TIF) [file pbio.3001569.s014.tif]

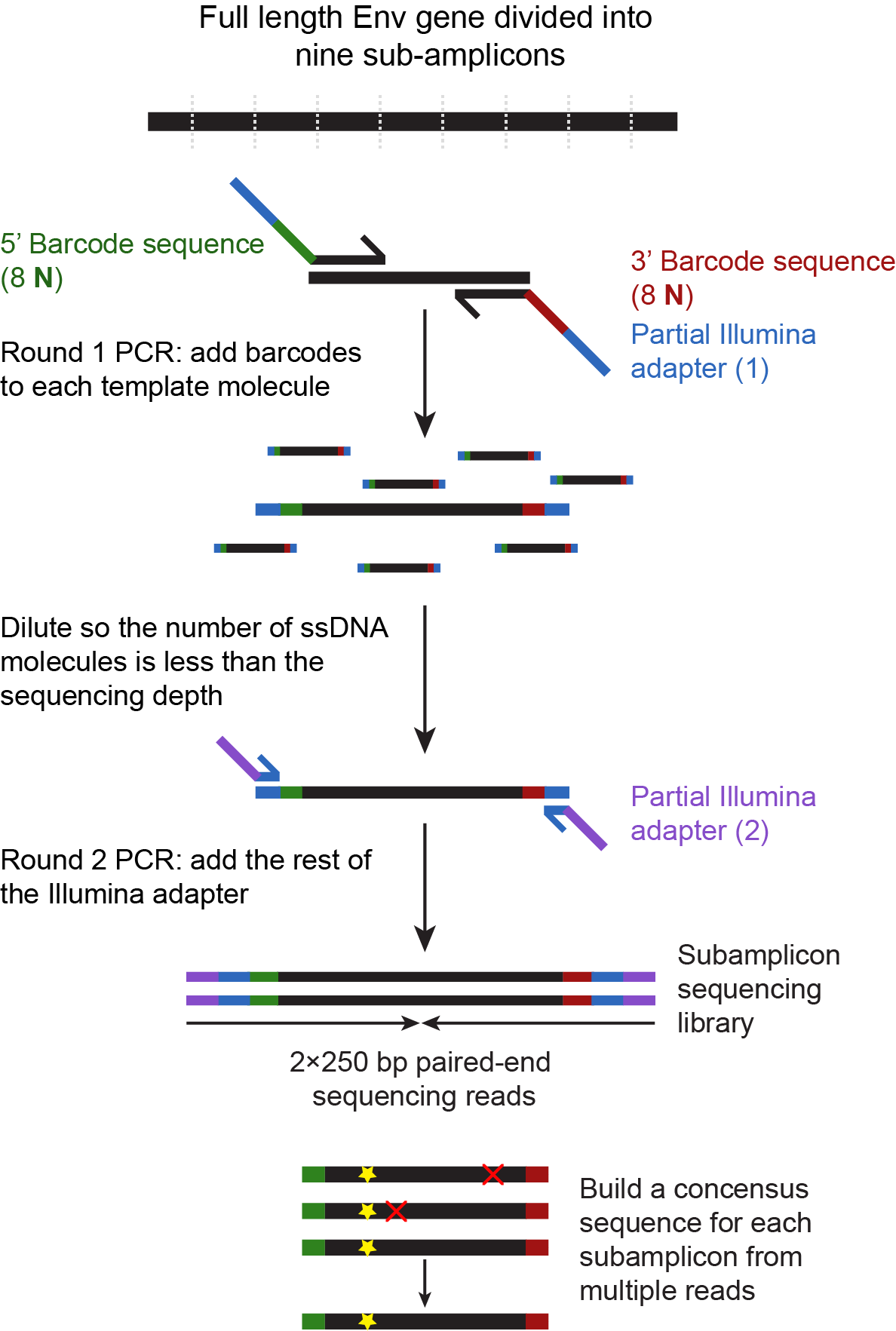

Supplement: S4 Fig — The full-length Env gene was divided into 9 subamplicons. In the first round of PCR, unique, random barcodes and part of the Illumina adapter were appended to the Env subamplicon molecules. In the second round of PCR, the complexity of the uniquely barcoded subamplicons was controlled to be less than the sequencing depth, and the remainder of the Illumina adapter was appended. The resulting libraries were sequenced on an Illumina HiSeq 2500 in rapid run mode with 2 × 250-bp paired-end reads. (TIF) [file pbio.3001569.s015.tif]

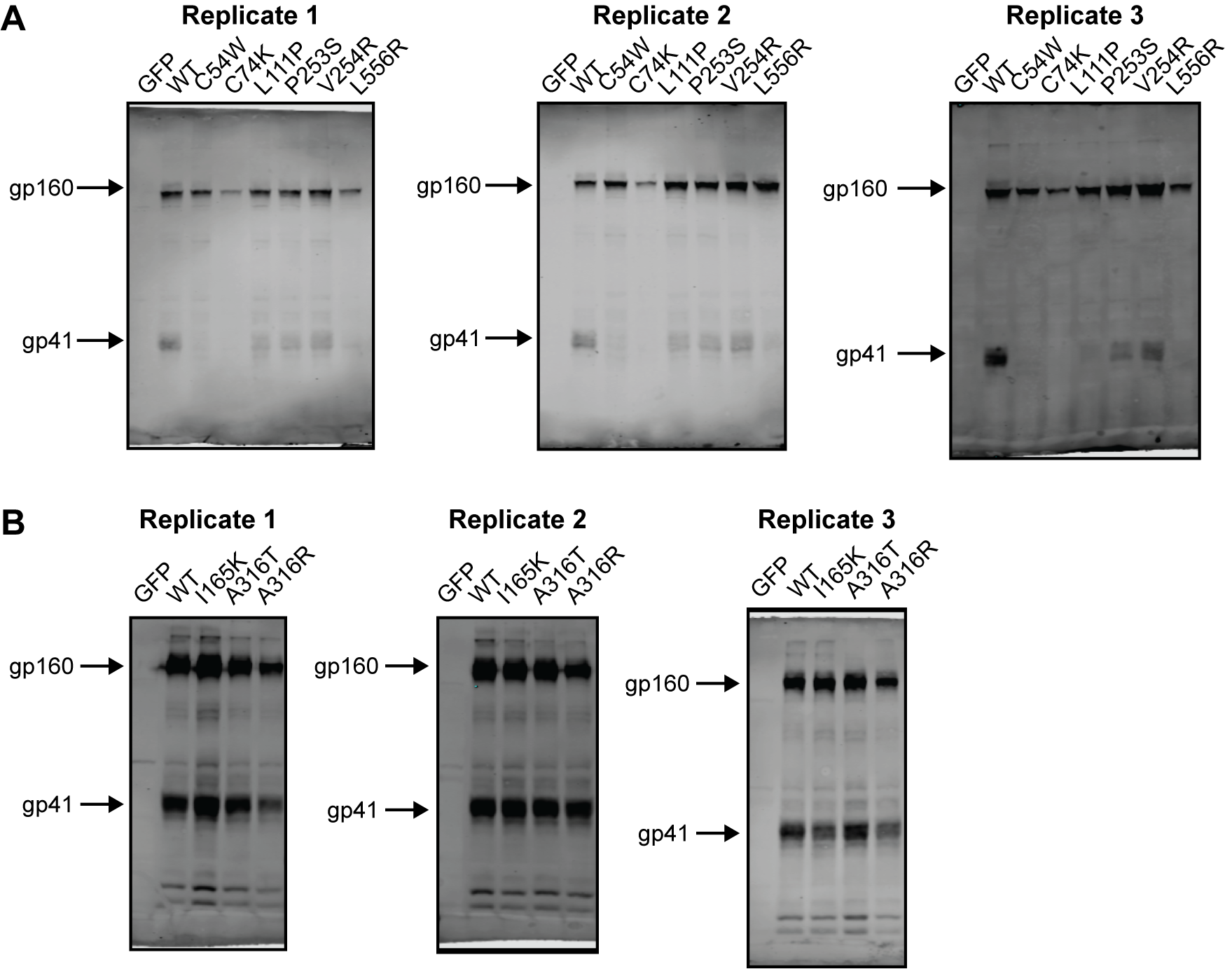

Supplement: S5 Fig — Immunoblots in biological triplicates showing gp160 and gp41 bands for selected variants with (A) negative diffsel and (B) positive diffsel upon XBP1s induction. (TIF) [file pbio.3001569.s016.tif]

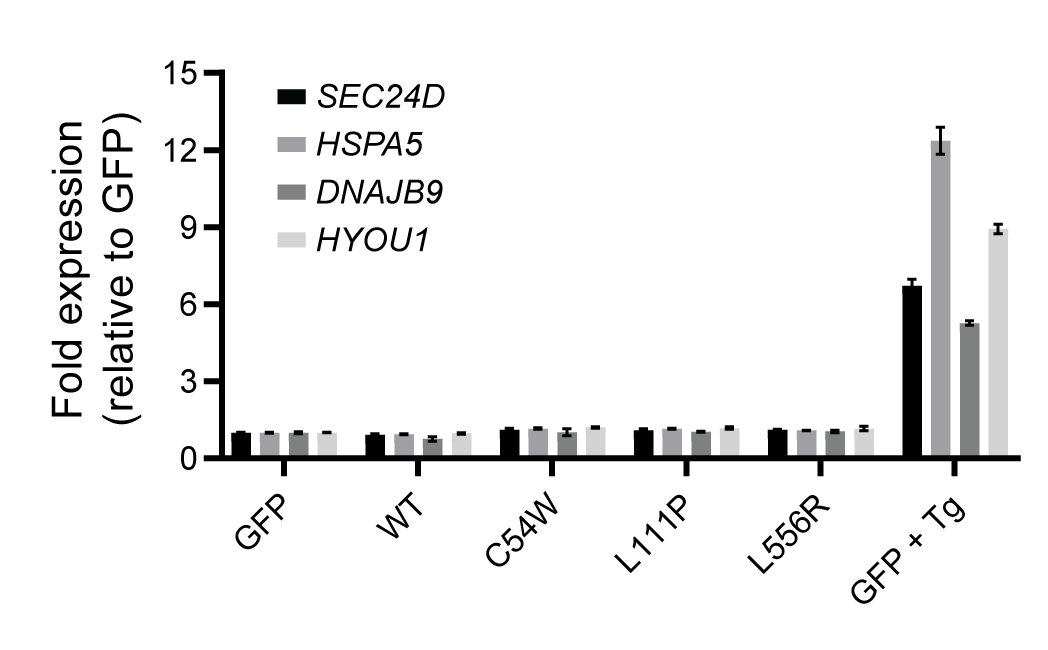

Supplement: S6 Fig — RT-PCR analysis of SEC24D, HSPA5, DNAJB9, and HYOU1 in HEK293T cells expressing GFP (negative control), wild-type Env, and 3 Env variants that were strongly negatively selected in +XBP1s versus basal (C54W, L111P, and L556R). As a positive control for UPR induction, HEK293T cells expressing GFP were treated with thapsigargin (Tg; 2 μM) for 6 h (GFP + Tg). RT-PCR data are presented as fold increase relative to GFP-transfected negative control. RT-PCR data values are provided in S7 Data. (TIF) [file pbio.3001569.s017.tif]

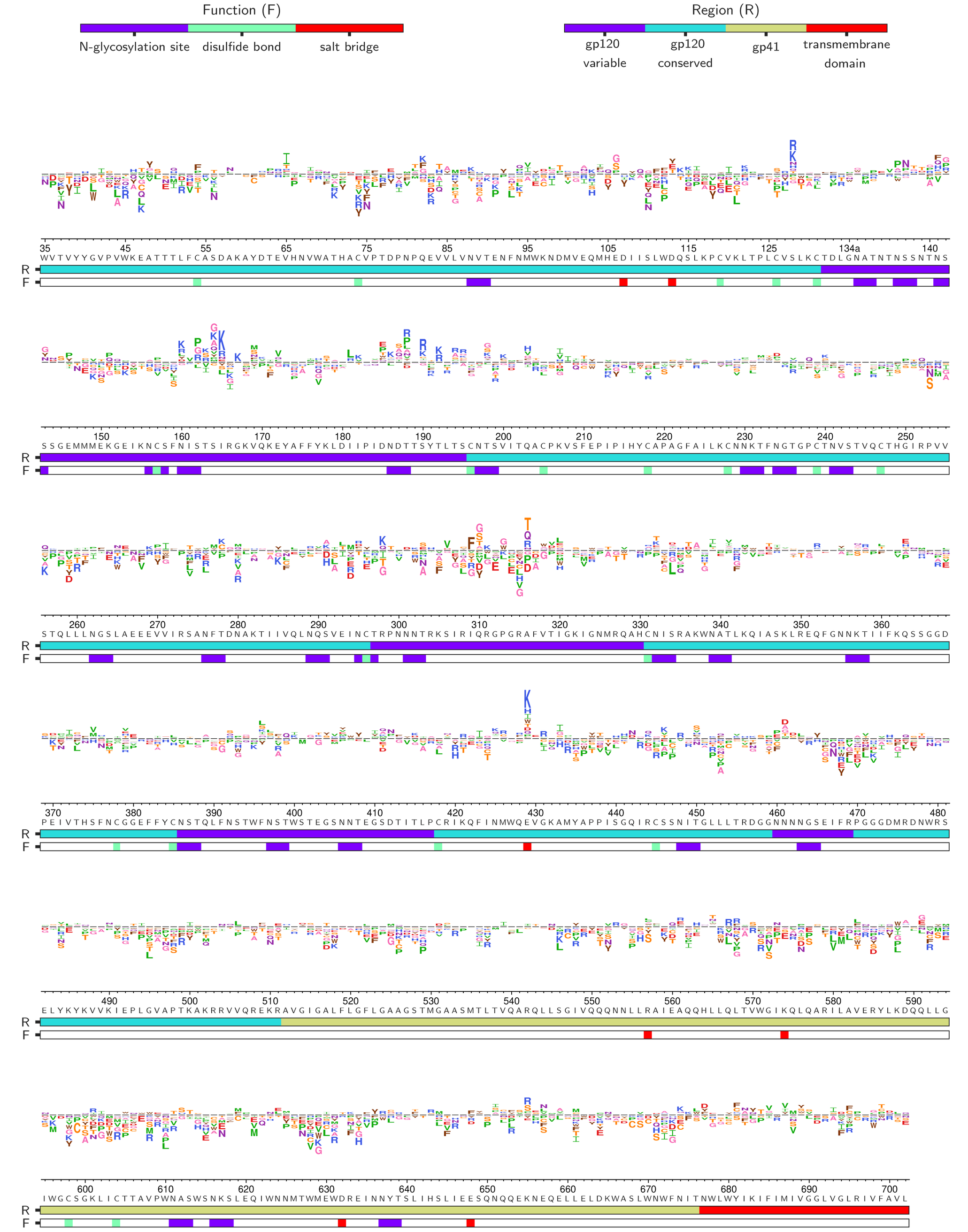

Supplement: S7 Fig — Logo plot displaying averaged diffsel for +XBP1s/+ATF6 normalized to the basal proteostasis environment. The height of the amino acid abbreviation corresponds to the magnitude of diffsel. The amino acid abbreviations are colored based on their side-chain properties: negatively charged (D, E; red), positively charged (H, K R; blue), polar uncharged (C, S, T; orange/N, Q; purple), small nonpolar (A, G; pink), aliphatic (I, L, M, P, V; green), and aromatic (F, W, Y; brown). The numbers and letters below the logos indicate the Env site in HXB2 numbering and the identity of the wild-type amino acid for that site, respectively. The color bar below the logos indicates the function (F) that the site is involved in (N-glycosylation site [purple], disulfide bond [green], or salt bridge [red]) or the region (R) of Env that the site belongs to (gp120–variable [purple], gp120–conserved [cyan], gp41 [yellow], or transmembrane domain [red]; the sites that belong to the 5 variable loops of gp120 were categorized as “gp120–variable,” and the sites that are not included in the 5 variable loops were categorized as “gp120–conserved”). Only variants that were present in all 3 pre-selection viral libraries and exhibited diffsel in the same direction across all 3 biological triplicates are plotted here. Diffsel values as well as unfiltered logo plots for each individual replicate are provided at https://github.com/yoon-jimin/2021_HIV_Env_DMS. (TIF) [file pbio.3001569.s018.tif]

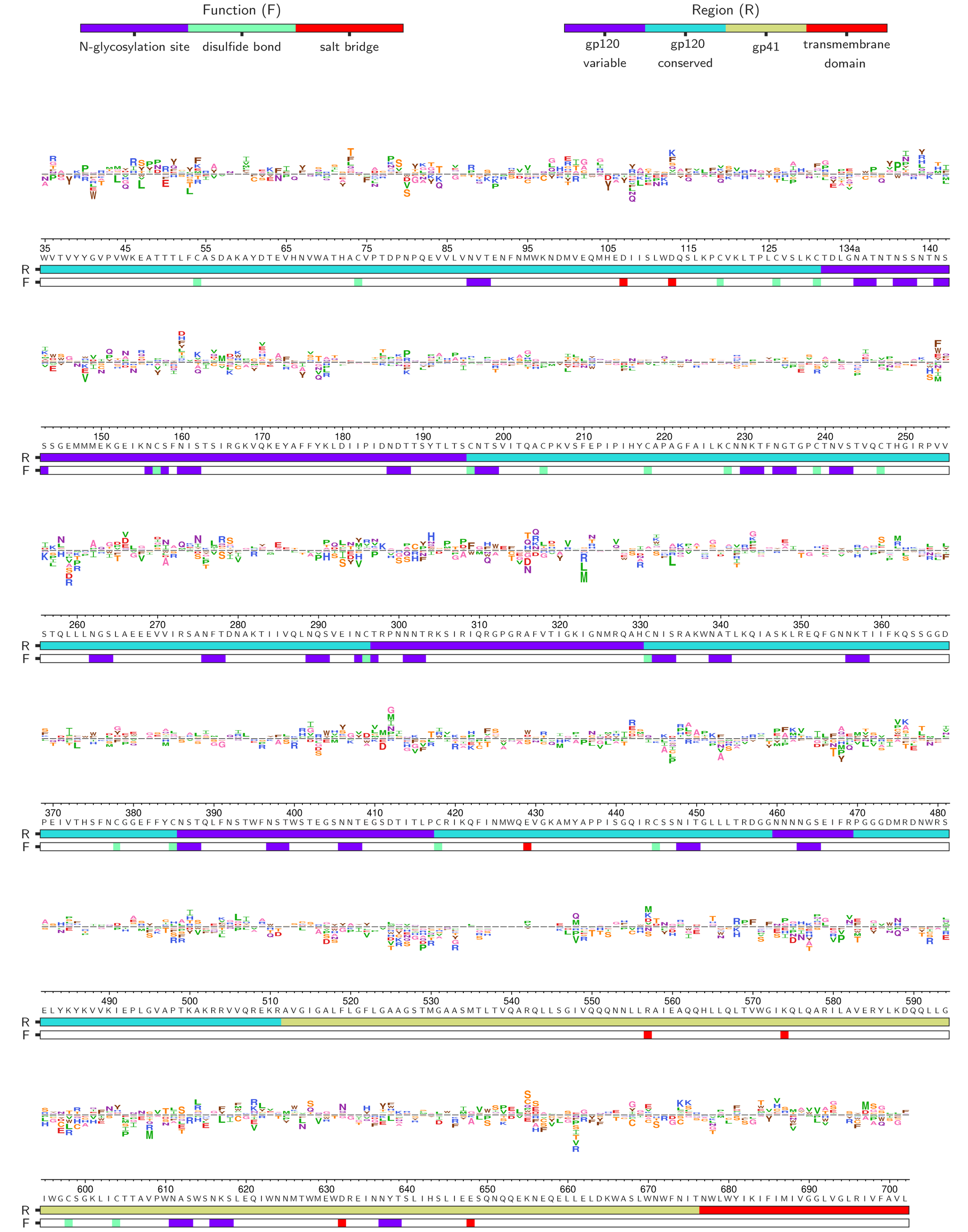

Supplement: S8 Fig — Logo plot displaying averaged diffsel for +ATF6 normalized to the basal proteostasis environment. The height of the amino acid abbreviation corresponds to the magnitude of diffsel. The amino acid abbreviations are colored based on their side-chain properties: negatively charged (D, E; red), positively charged (H, K R; blue), polar uncharged (C, S, T; orange/N, Q; purple), small nonpolar (A, G; pink), aliphatic (I, L, M, P, V; green), and aromatic (F, W, Y; brown). The numbers and letters below the logos indicate the Env site in HXB2 numbering and the identity of the wild-type amino acid for that site, respectively. The color bar below the logos indicates the function (F) that the site is involved in (N-glycosylation site [purple], disulfide bond [green], or salt bridge [red]) or the region (R) of Env that the site belongs to (gp120–variable [purple], gp120–conserved [cyan], gp41 [yellow], or transmembrane domain [red]; the sites that belong to the 5 variable loops of gp120 were categorized as “gp120–variable,” and the sites that are not included in the 5 variable loops were categorized as “gp120–conserved”). Only variants that were present in all 3 pre-selection viral libraries and exhibited diffsel in the same direction across all 3 biological triplicates are plotted here. Diffsel values as well as unfiltered logo plots for each individual replicate are provided at https://github.com/yoon-jimin/2021_HIV_Env_DMS. (TIF) [file pbio.3001569.s019.tif]

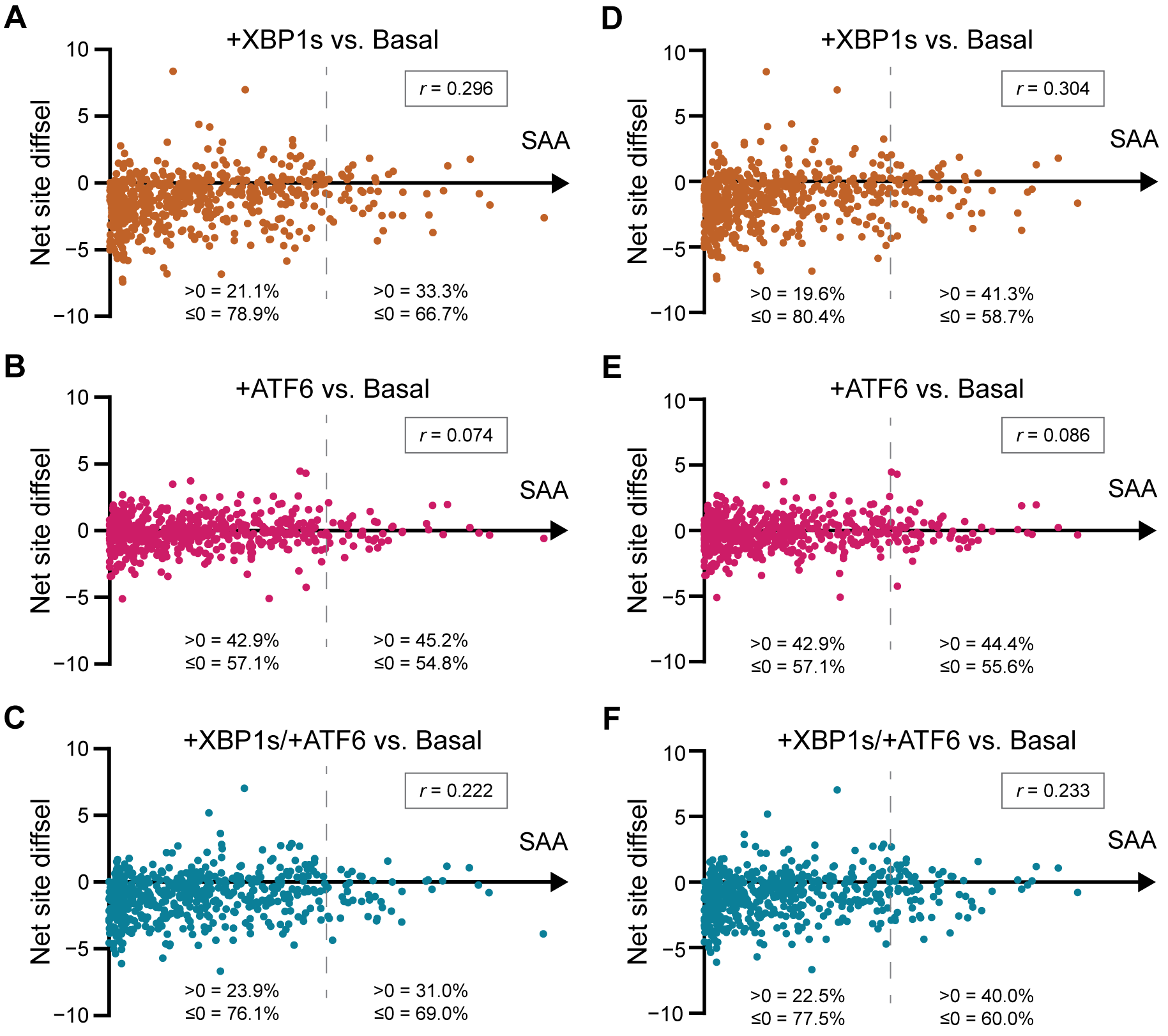

Supplement: S9 Fig — Average net site diffsel values plotted against the SAA of Env monomer (A–C) and trimer (D–F). Average net site diffsel values for +XBP1s (A and D), +ATF6 (B and E), and +XBP1s/+ATF6 (C and F) were normalized to the basal ER proteostasis environment and plotted against the SAA at each site. The percentages of variants with positive and negative net site diffsel for the left and right half of the plot are stated, as well as the Pearson correlation coefficient r. SAA was calculated using PDBePISA [94] with PDB ID 5V8M [95], where SAA = 0 corresponds to a buried site. SAA data values are provided in S9 Data. (TIF) [file pbio.3001569.s020.tif]

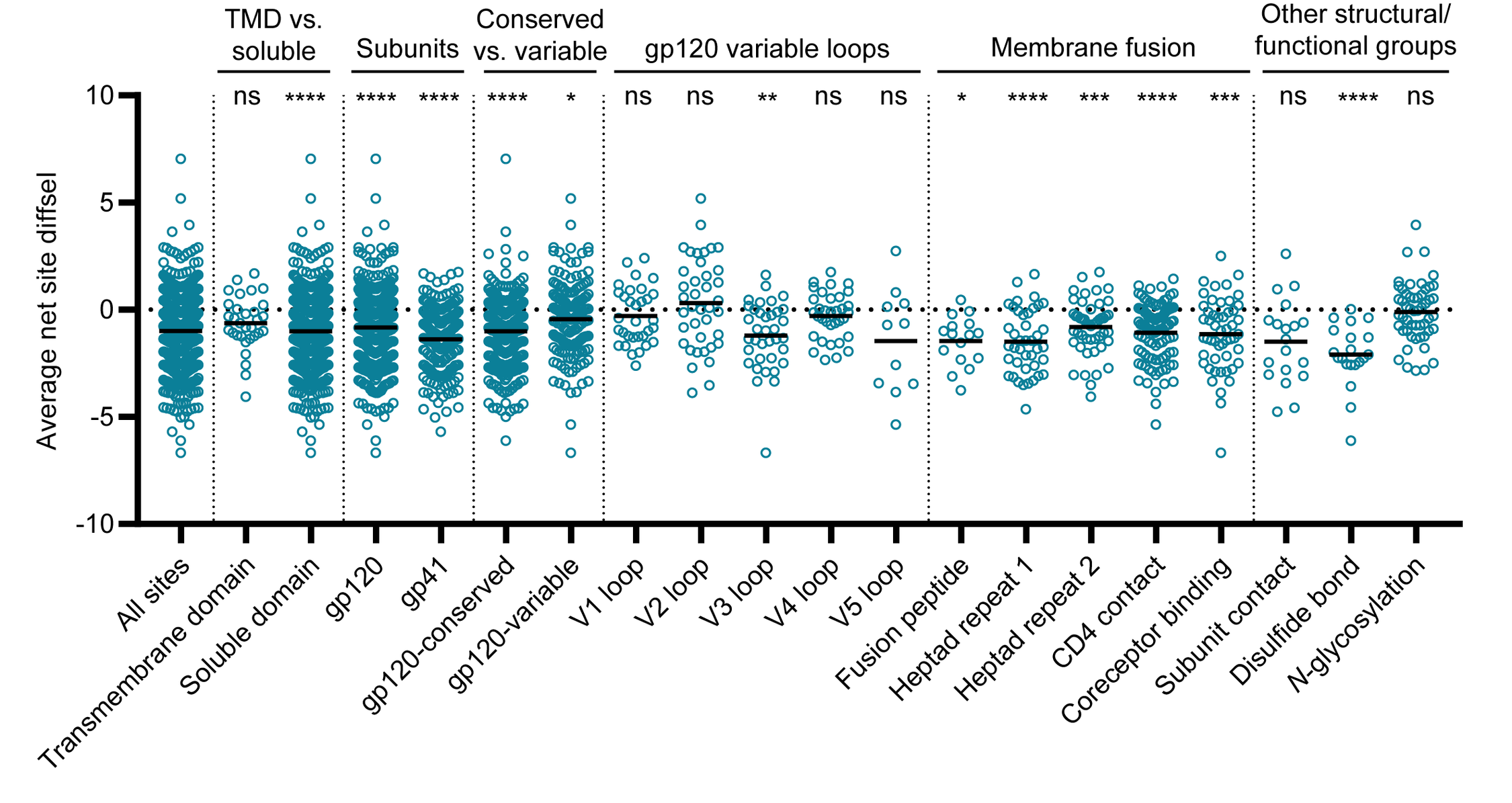

Supplement: S10 Fig — Average net site diffsel for the +XBP1s/+ATF6 ER proteostasis environment normalized to the basal ER proteostasis environment, where the means of distributions are indicated by black horizontal lines. Sites are sorted by TMD versus soluble, subunits, conserved versus variable regions of gp120, the 5 variable loops of gp120, regions important for membrane fusion, and other structural/functional groups. For TMD versus soluble, all sites that do not belong to the TMD were categorized as “soluble.” For conserved versus variable, the sites that belong to the 5 variable loops of gp120 were categorized as “gp120–variable,” and the sites that are not included in the 5 variable loops were categorized as “gp120–conserved.” Significance of deviation from null (net site diffsel = 0, no selection) was tested using a 1-sample t test. The derived p-values were Bonferroni-corrected for 20 tests;*p-value < 0.05, **p-value < 0.01, ***p-value < 0.001, ****p-value < 0.0001; ns, not significant. Diffsel values are provided at https://github.com/yoon-jimin/2021_HIV_Env_DMS. Assignments for these structural regions are provided in S2 Table. (TIF) [file pbio.3001569.s021.tif]

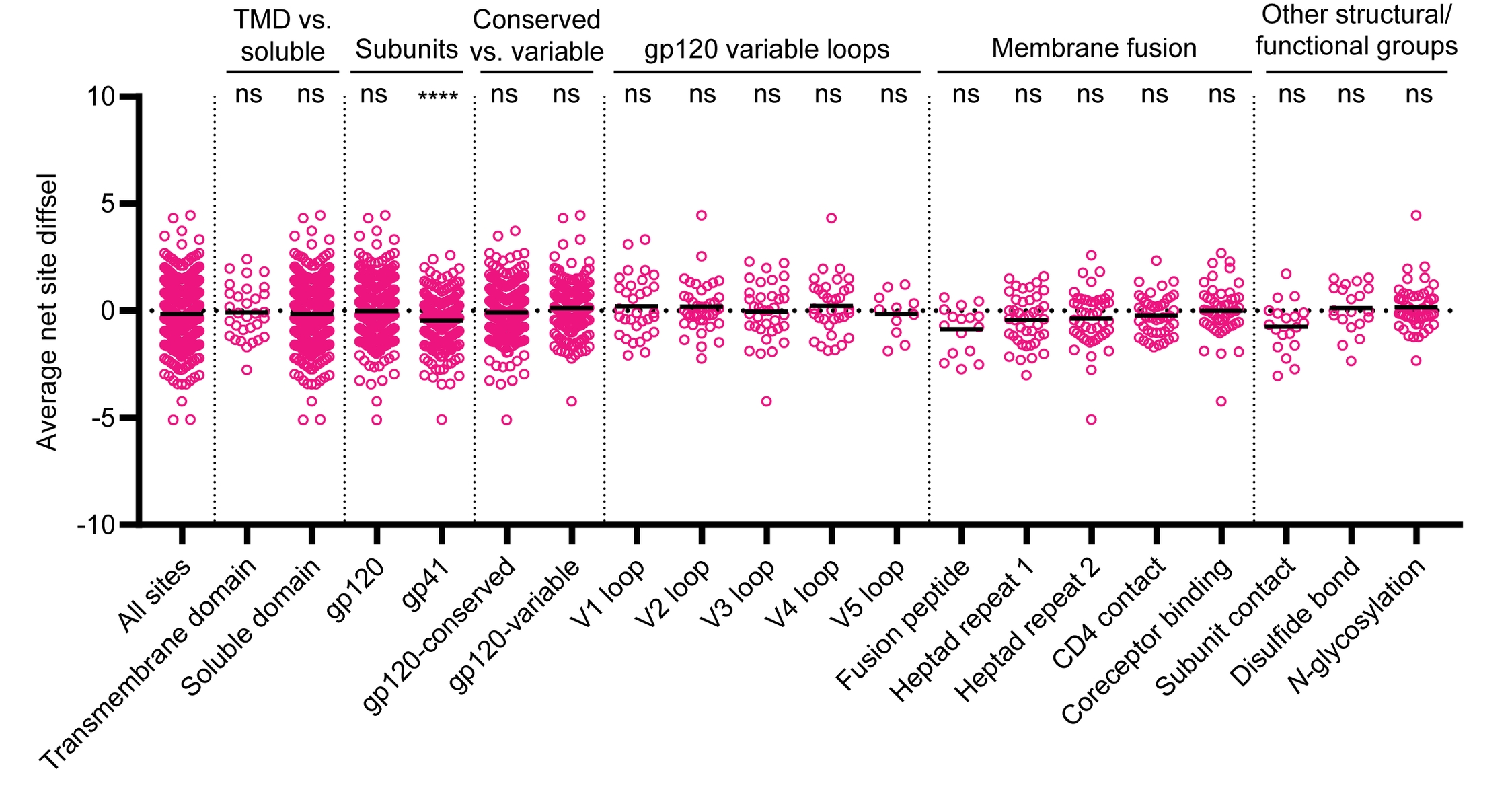

Supplement: S11 Fig — Average net site diffsel for the +ATF6 ER proteostasis environment normalized to the basal ER proteostasis environment, where the means of distributions are indicated by black horizontal lines. Sites are sorted by TMD versus soluble, subunits, conserved versus variable regions of gp120, the 5 variable loops of gp120, regions important for membrane fusion, and other structural/functional groups. For TMD versus soluble, all sites that do not belong to the TMD were categorized as “soluble.” For conserved versus variable, the sites that belong to the 5 variable loops of gp120 were categorized as “gp120–variable,” and the sites that are not included in the 5 variable loops were categorized as “gp120–conserved.” Significance of deviation from null (net site diffsel = 0, no selection) was tested using a 1-sample t test. The derived p-values were Bonferroni-corrected for 20 tests; ****p-value < 0.0001; ns, not significant. Diffsel values are provided at https://github.com/yoon-jimin/2021_HIV_Env_DMS. Assignments for these structural regions are provided in S2 Table. (TIF) [file pbio.3001569.s022.tif]

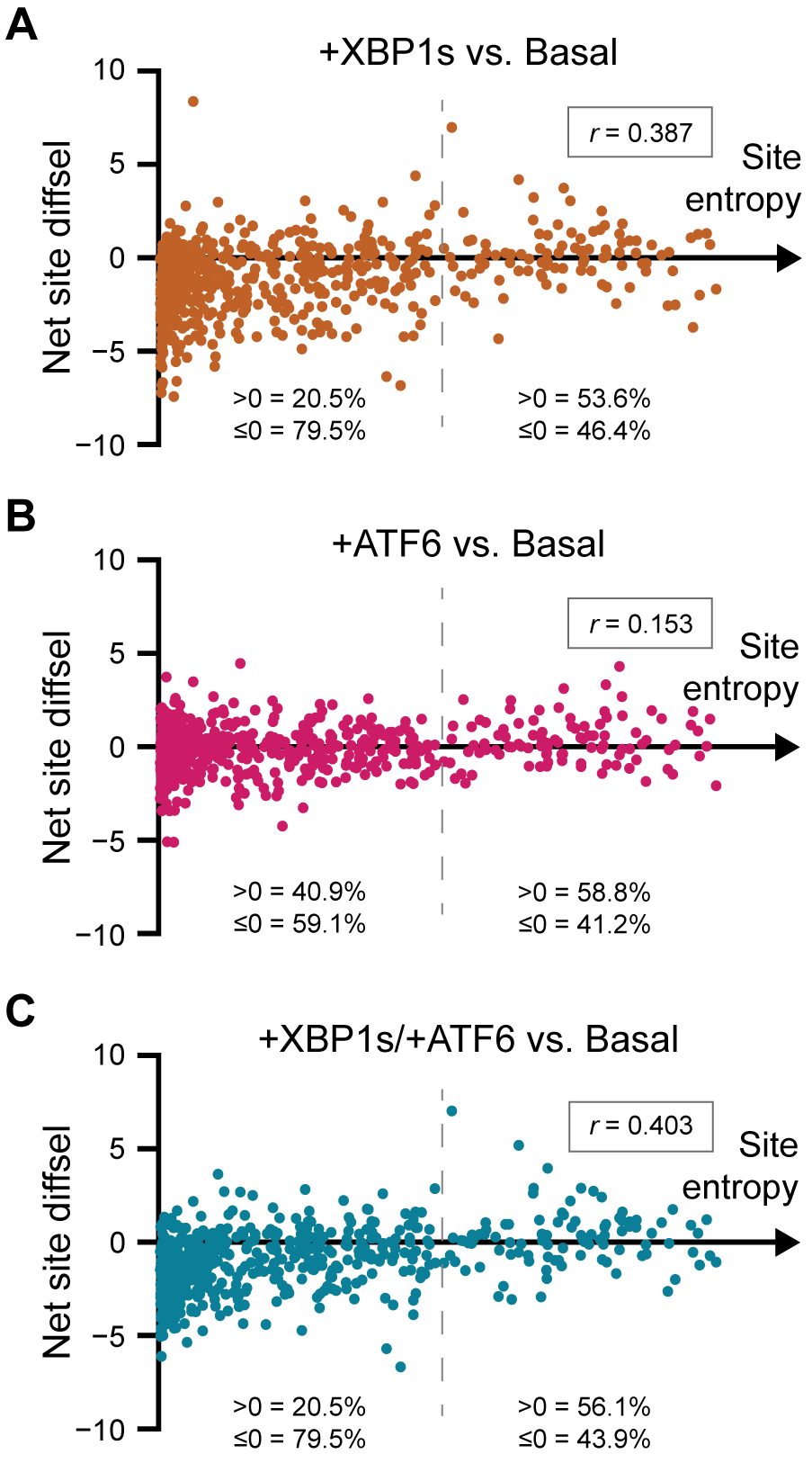

Supplement: S12 Fig — Average net site diffsel values across Env for (A) +XBP1s (B) +ATF6, and (C) +XBP1s/+ATF6 are normalized to the basal ER proteostasis environment and plotted against the site entropy at each site. The percentages of variants with positive and negative net site diffsel for the left and right half of the plot are stated, as well as the Pearson correlation coefficient r. Site entropy data values are provided in S10 Data. (TIF) [file pbio.3001569.s023.tif]

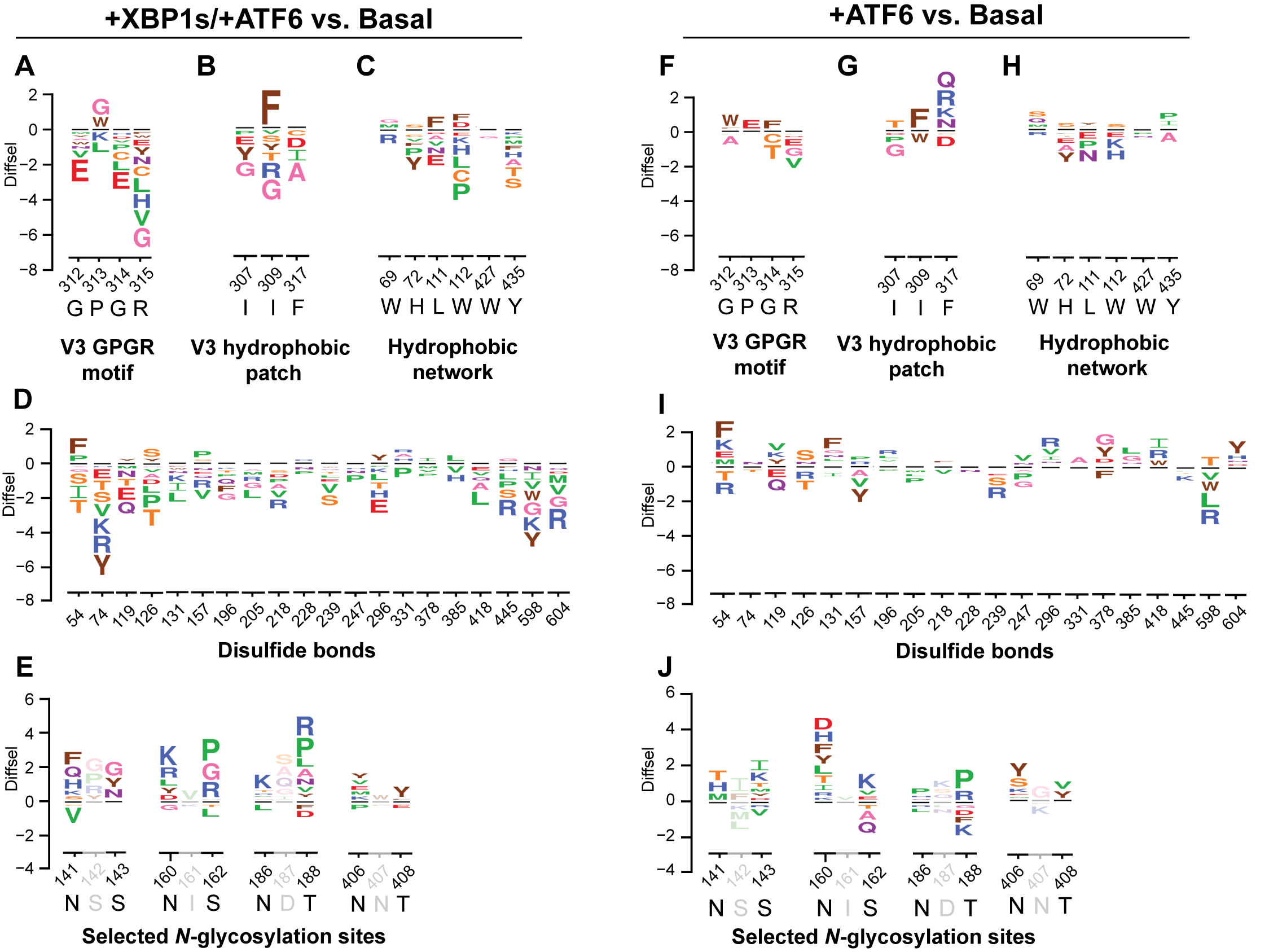

Supplement: S13 Fig — Selected sequence logo plots for the +XBP1s/+ATF6 (A–E) and +ATF6 (F–J) ER proteostasis environments normalized to the basal ER proteostasis environment for (A and F) the conserved GPGR motif of the V3 loop, (B and G) the hydrophobic patch of the V3 loop, (C and H) the hydrophobic network of gp120 (important for CD4 binding), (D and I) cysteine residues participating in disulfide bonds, and (E and J) selected N-glycosylation sequons (N-X-S/T) that exhibited positive net site diffsel in all 3 remodeled proteostasis environments. The height of the amino acid abbreviation corresponds to the magnitude of diffsel. The numbers and letters below the logos indicate the Env site in HXB2 numbering and the wild-type amino acid for that site, respectively. Only variants that were present in all 3 pre-selection viral libraries and exhibited diffsel in the same direction across the biological triplicates are plotted. All logo plots were generated on the same scale. Diffsel values are provided at https://github.com/yoon-jimin/2021_HIV_Env_DMS. Assignments for these functional regions are provided in S2 Table. (TIF) [file pbio.3001569.s024.tif]

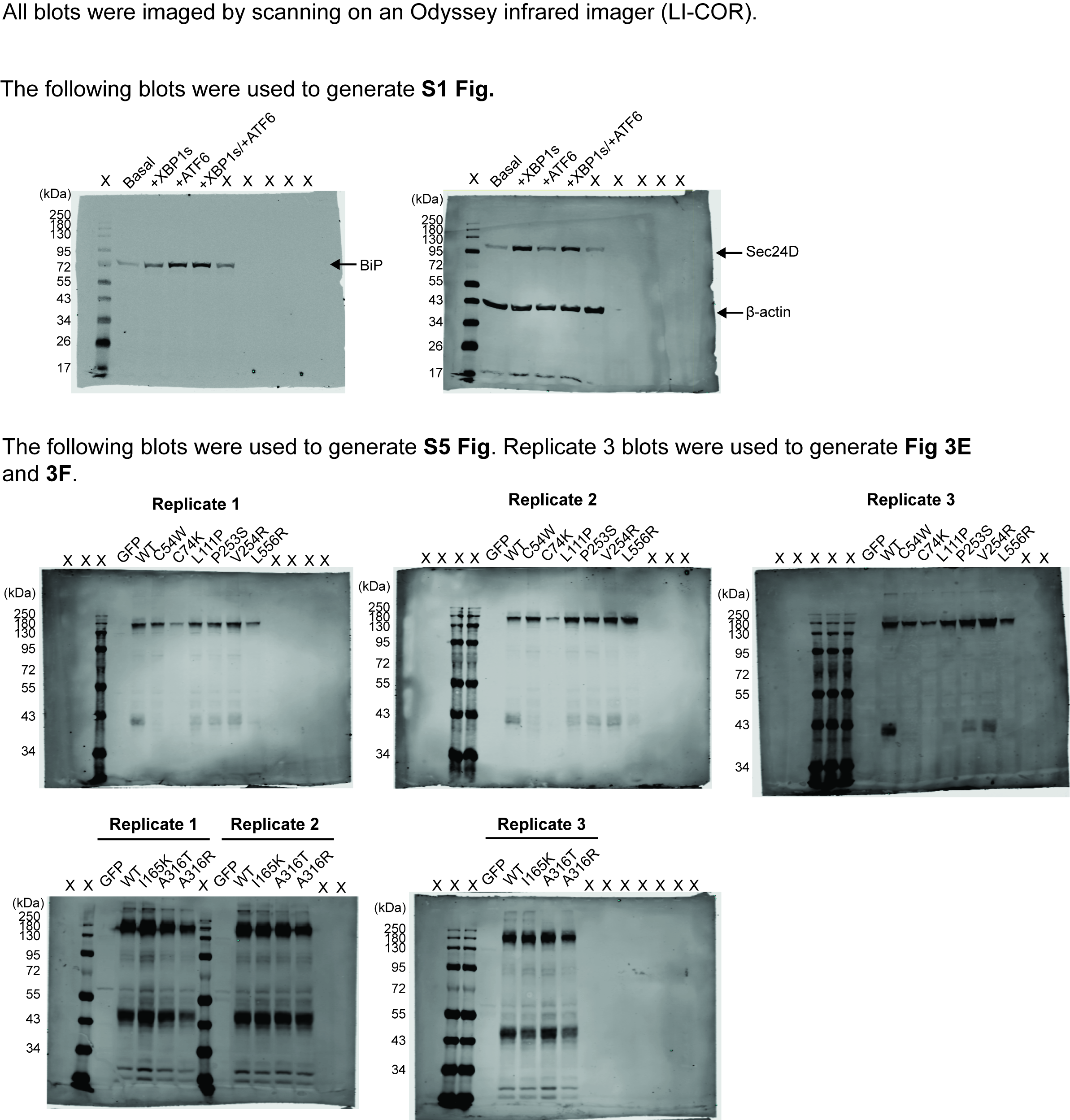

Supplement: S1 Raw Images — (TIF) [file pbio.3001569.s025.tif]
